# Neural encoding of native and non-native speech sounds in quiet and in background noise

**DOI:** 10.3389/fnins.2026.1823312

**Published:** 2026-05-26

**Authors:** Reethee Antony, Brett Martin, Valerie Shafer, Susan Behrens

**Affiliations:** 1 Department of Speech-Language-Hearing Sciences, The Graduate Center, City University of New York (CUNY), New York, NY, United States; 2 Division of Speech and Language Pathology, Binghamton University, State University of New York (SUNY), New York, NY, United States; 3 Department of Communication Sciences, and Disorders, Marymount Manhattan College, New York, NY, United States

**Keywords:** cortical auditory evoked potentials, neural encoding, neurophysiologic processing, non-native speech perception, speech processing

## Abstract

**Background:**

The neural encoding of voicing in speech sounds has been relatively well studied in monolingual, native speakers. Little research, however, has examined neural encoding of aspiration feature in speech sounds or focused on encoding of non-native phonetic features or the effect of noise on processing these features.

**Purpose:**

This study examined Auditory Evoked Potentials (AEPs) to bilabial stops with English versus Hindi phonetic properties of aspiration and voicing Hindi, English and Tamil listeners, in quiet and in noise.

**Method:**

A total of 48 participants (16 Hindi, 16 American English, and 16 native Tamil native speakers) between 20 and 45 years of age participated. Natural digitized speech sounds including Hindi /ba/, /pa/, and /p^h^a/ and American English /ba/, and /pa/ were presented at 70 dB SPL using insert earphones in quiet, and in background noise at signal-to-noise ratio of 0. AEP peaks P1, N1, P2, and N2 were measured at the central electrode site (FCz).

**Results:**

The P1 and P2 peak amplitudes were significantly larger for Hindi CV stimuli in Hindi participants relative to American English participants and Tamil participants. The morphology to Hindi /p^h^a/ was similar to English /pa/, and the morphology for Hindi /pa/ was similar to English /ba/. P1 amplitudes were larger and P2 amplitudes were smaller in noise relative to quiet. N2 peak latency in response to Hindi /pa/ was slightly longer relative to Hindi /p^h^a/ in American English listeners.

**Conclusion:**

The findings add evidence to the Automatic Speech Perception model by observing cross-linguistic differences in P1 and P2. The results contribute to a better understanding of neural encoding in the cortex across native and non-native listeners, and how noise modulates early stages of processing.

## Background

Processing of speech sounds can be studied using various methods. Auditory evoked potentials (AEPs) are neural responses that are elicited by sound stimuli and are generated in/near the auditory cortex (Davis, 1939; Davis et al., 1967). A simple speech stimulus (e.g., CV stimuli or V stimuli) elicits a sequence of positive and negative obligatory peaks, P1-N1-P2, that index encoding of auditory information. This P1-N1-P2 complex can be described in terms of polarity, amplitude and latency (Näätänen and Picton, 1987; Ceponiene et al., 1998; Ponton et al., 2002). The underlying sources of this complex of peaks can be dissociated (Näätänen and Picton, 1987). P1 and P2 are vertex positive peaks with latencies around 50 ms and 180 ms post stimulus onset, respectively, to simple auditory stimuli, such as tones (Geisler et al., 1958; Hyde, 1997; Picton, 2013). N1 is a vertex negative peak with a latency of approximately 100 ms (Hyde, 1997; Picton, 2013). There are several (at least 3) sub-components to N1 with peak latencies around 70–150 ms post stimulus onset (Näätänen and Picton, 1987; Näätänen et al., 2004; Giard et al., 1994; Woods, 1995; Picton, 2013). These sub-components can be teased apart by parametric manipulations and by examining topography. An additional peak, the N2, is a vertex negative peak with latency around 200 ms post-stimulus onset. The N2 is not always present in adults in passive paradigms (without directed attention), and is modulated by task. Thus, it may serve as an indication of attentional allocation to a stimulus.

Many studies using AEPs have revealed that these measures reflect encoding of the acoustic features of speech sounds at a fine-grained level (e.g., Martin et al., 1997, 1999; Tremblay, 2005; Whiting et al., 1998; see Martin et al., 2008 for review).

Few studies have systematically examined whether AEPs are sensitive to language-specific patterns (e.g., Elangovan and Stuart, 2011; Jayakumar and Narne, 2021; Sharma and Dorman, 2000). Several of the studies have observed that AEPs reflect categorical boundaries (e.g., Bidelman et al., 2013; Sharma and Dorman, 1999; Sharma et al., 2000) and are sensitive to spectra-temporal speech properties (Toscano et al., 2010; Wagner et al., 2013). For example, Bidelman et al. (2013) found that the amplitude of P2 reflected categorical perception (vowel /u/ vs. /a/), whereas Sharma and colleagues observed two N1 peaks for long-lag voice-onset times (VOTs), greater than 30 ms for /ba/−/pa/ and greater than 40 ms for /da/−/ta/ and /ga/−/ka/ continua in American-English listeners (Sharma and Dorman, 1999; Sharma, et al., 2000). These findings of sensitivity of AEPs to categorical boundaries suggest that language experiences should modulate AEPs. However, the few studies directly examining P1-N1-P2 in cross-linguistic studies have not observed differences related phonological status. For example, Sharma and Dorman (2000) found no difference in N1 to Hindi prevoiced versus short-lag stops between Hindi and English listeners. In contrast, the Mismatch Negativity (MMN) discriminative response, elicited in an oddball paradigm is modulated by native-language experiences, as shown by multiple studies (e.g., Dehaene-Lambertz et al. 2000; Näätänen and Picton, 1987; Näätänen et al., 2004; Sharma and Dorman, 2000; Shafer et al. 2021). These findings have been used to suggest that AEPs are sensitive to spectral-temporal speech patterns at an acoustic phonetic level, and it is only at higher-levels of neural processing, indexed by the MMN and later, endogenous components (e.g., P3b) that phonological status modulates processing.

Previous studies have only examined AEPs of speech sounds in quiet contexts, in addition to the sparsity of research that has focused on cross-linguistic studies of AEPs (with most studies examining MMN). For these reasons, we examine AEPs in a cross-linguistic design to more fully examine the claim that AEPs are not sensitive to phonological factors.

### Theoretical frameworks

Many studies have demonstrated speech perception difficulty for non-native speech (e.g., Best et al., 1988; Best, 1995; Flege, 1988). For the current study, two models related to cross-linguistic processing of speech in noise were relevant. In the Automatic Selective Perception *(ASP) Model* (Strange and Shafer, 2008; Shafer, 2024), speech identification and discrimination of native sounds is rapid and robust in suboptimal listening conditions, even when listeners focus on other levels of language structure, or on another task. Non-native listeners of Second Language (L2) listeners more perform well under optimal listening conditions, but when conditions are suboptimal, such as in noise, performance often suffers.

The current study derives hypotheses form the ASP model. The theoretical question of interest was whether non-native or L2 speakers of a language show less robust processing of speech cues, particularly in noise. This study will further test the ASP model, providing information on whether speech processing at the level of AEPs is shaped by native-language input, and provide further information on the relative difficulty of voicing versus aspiration cues.

The second relevant model is the Perceptual Assimilation Model (PAM-L2; Best et al., 1988; Best, 1995; Best and Tyler, 2007). In this model, non-native speech sound perception can be explained using four mechanisms. A non-native speech sound, such as /b^h^/ for English listeners, can be perceptually assimilated as a categorized exemplar of /b/. Alternatively, two non-native speech sounds can be categorized as the same sound with one being a better exemplar than the other. For example, Hindi /b/ and /b^h^/ could be perceived as /b/, with /b/ being a better exemplar relative to /b^h^/. Further, a non-native sound can be perceived as an uncategorized consonant that has similarity to two different native categories. For example, a Hindi listeners might categorize a non-native speech sound such as /ɵ/, as somewhat like a dental /t/ and somewhat like the labiodental fricative /f/. Lastly, if a non-native speech sound does not have any resemblance to any of the native phonemes, new categories can be formed, such as a Zulu click for a native English listener.

PAM-L2 was used in this study to predict the patterns of perceptual assimilation that take place in non-native listeners to Hindi versus American English speech sounds. The Hindi participants in this study were non-native listeners of American English speech sounds, the English participants were non-native listeners of Hindi speech sounds, and the Tamil listeners were non-native listeners of both Hindi and English speech sounds.

### AEP studies of stop consonants

Hindi is an Indo-Aryan language and has been less studies than European languages, such as English and German. Most previous work that has examined the neural processing of Hindi speech sounds in native and non-native listeners has focused on Hindi retroflex consonants (e.g., Chen and Small, 2015; Dehaene-Lambertz, 1997; Rivera-Gaxiola et al., 2000; Shafer et al., 2004; Small and Werker, 2012). Hindi, like English, makes contrastive use of voicing and aspiration features (Bhaskararao, 2011), but the phonetic details differ between the two languages. In general, studies show that the AEP peak latencies are more prolonged with increases in VOT (from short-lag to longer-lag times between the stop consonant burst and onset of voicing), thereby reflecting the stimulus characteristics (Elangovan and Stuart, 2011; Han et al., 2013; Sharma and Dorman, 2000; Toscano et al., 2010). English voiceless stops are also aspirated, but aspiration is not contrastive (that is, not phonemic), and serves as a secondary cue.

As mentioned previously, two studies observed two N1 peaks for long-lag VOT consonants, /pa/, /ta/ and /ka/ for American English listeners (Sharma and Dorman, 1999; Sharma et al., 2000). In these studies, VOT was varied systematically (increasing from 0 ms in 10-ms steps) by editing natural speech to produce a continuum. For English listeners, these stimuli cross a phonemic boundary (from short-lag VOT, /ba/, /da/ and /ga/ to long-lag VOT, /pa/, /ta/ and /ka/, respectively). The presence of the additional N1 peak was observed only to the stimuli behaviorally categorized as long-lag VOT phonemes.

In an additional study, Sharma and Dorman (2000), tested whether N1 was sensitive to categorical perception for a prevoiced VOT-short lag VOT contrast which is phonemic in Hindi, but not in English. Stimuli consisted of 10 ms steps from −90 ms prevoicing to 0 ms prevoicing. The results revealed that, perceptually, American English speakers were poor at categorizing the stimuli (perceiving all stimuli as members of the /ba/ category), whereas Hindi speakers showed a category boundary at approximately −30 ms, perceiving stimuli with −40 to −90 VOTs as Hindi /ba/, and those with 0 to −20 VOTs as Hindi /pa/. In contrast, at the level of the N1 latency, there was no group difference between Hindi and English listeners. The possible presence of a second N1 peak for prevoicing across the category boundary (−40 to −90 ms) was not addressed in this paper. The double N1 peaks for short-lag versus long-lag VOT stops have been attributed to the differences in acoustic properties of the stimuli in terms of VOT, burst duration, amplitude of aspiration, and F1 height (Sharma et al., 2000; Steinschneider, 2004).

Except for the papers by Sharma and colleagues, most previous work that has examined the neural processing of Hindi speech sounds in non-native listeners has focused on Hindi retroflex consonants (e.g., Chen and Small, 2015; Dehaene-Lambertz, 1997; Rivera-Gaxiola et al., 2000; Shafer et al., 2004; Small and Werker, 2012). The most relevant to the study of AEPs is Small and Werker (2012), who examined Acoustic Change Complex (ACC) responses to Hindi speech sounds that differed in place of articulation (bilabial /b/, dental /d/, and retroflex /D/) using the stimuli /daba/, /dada/, and /daDa/ in native English listeners. The ACC reflects the P1-N1-P2 from the change point, in this case, the second syllable. The ACC P1-N1-P2 peak amplitude responses to /daba/ were larger than the responses to /dada/ and /daDa/. However, the absence of a native Hindi group in this study does not allow distinguishing between a phonetic and a phonological explanation for the findings. In addition, for AEPs, the spectral differences reflected by place of articulation are more subtle than temporal differences of VOT.

To fully test whether AEPs are modulated by language specific experiences, it is critical to include both a native and non-native group (e.g., Sharma and Dorman, 2000). Elangovan and Stuart (2011) examined 10 native English speakers and 10 native Spanish speakers using a /ba/−/pa/ continuum that varied in voice onset time (VOT) from 0 to 60 ms. The mean categorical boundary was longer for the English group (mean = 26.8 ms; SD = 6.1 ms) relative to the Spanish group (mean = 14.1 ms; SD = 4.6 ms). However, no significant difference was observed between the groups in terms of P1, N1, P2 latencies or P1-N1 amplitudes and N1-P2 amplitudes. The authors claim that the P1-N1-P2 potentials do not reflect linguistic specific phonetic boundaries. This study, along with Sharma and Dorman (2000) is important for cross-linguistic AEP research and provides evidence for a dissociation between behavioral findings (as evidenced from categorial boundary) and early cortical encoding.

There are languages where neither voicing nor aspiration are contrastive or phonemic. For example, in Tamil, a Dravidian language, voicing of consonants are non-contrastive, only serving as allophonic features. For example, Jayakumar and Narne (2021) compared AEPs of 10 Kannada speaking adults (20–35 years) with 10 Tamil speaking adults in the same age range using a /da/−/ta/ 10-step continuum. Kannada is a language with both voicing and aspiration whereas Tamil has no voicing or aspiration differences that are phonemic in nature. The authors found no significant group differences in N1 latency across the two language groups, contributing to the other reports that language-specific experience does not modulate N1.

The three crosslinguistic studies all tested relatively small samples with only 10 speakers for each language group and focused on stimuli that were native from one group and non-native for another. In addition, the studies focused on different AEP measures, with two examining only N1 latency and one focusing on P1-N1-P2 amplitudes. Thus, it is of interest to further examine whether there are native-language experience effects on AEPs.

### AEP studies of aspirated speech sounds

In general, there has been little work examining the neurophysiologic processing of aspiration. One study used the Korean language to examine N1-P2 amplitudes to aspirated stops (Han et al., 2013). The other study focused on musician and non-musician French speakers’ discrimination of voicing and aspiration cues that are phonemic in Thai but not French; they used the endogenous N200 (N2b) and P300 (P3b) components in an active discrimination task (Dittinger et al., 2018). Musician compared to non-musicians showed larger N2b and P3b to the non-native /p/ versus /ph/ contrast, but no difference for the native /b/ versus /p/ contrast. However, this study did not compare the findings to native, Thai speakers.

In another study, Bloder et al. (2024) examined German monolingual and Italian-German bilingual children neural processing and discrimination of Hindi /ba/, /pa/, and /p^h^a/. German contrasts short-lag and long-lag, aspiration stops (phonetically, [pa] vs. [p^h^a]), whereas Italian contrasts short-lag and voicing lead stops (phonetically, [ba] vs. [pa]). The Italian-German bilingual children showed an attenuated mismatch response compared to the monolingual German children, but only for the long-lag contrast closer to the adult category boundary (36 ms, VOT). Neither of these studies, however, examined obligatory frontal-central peaks. Bloder et al. (2024) found an attenuated T-complex response in the bilingual children over the lateral site (T8) to the 0-ms VOT, but no group difference for aspirated or voicing lead. In addition, they observed effects of the stimulus.

The one study that did focus on frontocentral N1-P1 that included an aspirated stop was Han et al. (2013). They examined neural processing of Korean aspirated, tense and lax stop consonants in a CV context. Results included larger N1-P2 amplitudes to aspirated /t^h^a/ and /p^h^a/ stops compared to their tense and lax counterparts. This finding suggests that there may be a potential neural signature for the Hindi aspirated speech sound /p^h^a/ used in the current study, as well.

### AEP studies of speech in noise processing

In the present study, the encoding of both voicing and aspiration features was examined in quiet and in noise. There is a considerable amount of work related to P1, N1, P2 to speech sounds in noise, but not focusing on Hindi contrasts or aspirated stops (e.g., Dimitrijevic et al., 2013; Martin et al., 1997; Martin et al., 1999; Martin and Stapells, 2005; Whiting et al., 1998). Overall, AEP responses are smaller in amplitude and the latencies are more prolonged in noise relative to quiet (e.g., Dimitrijevic et al., 2013; Martin et al., 1997, 1999; Martin and Stapells, 2005; Whiting et al., 1998). The impact of noise on AEP waveforms depends on many factors, including noise intensity, signal to noise ratio (SNR), spectral content, type of noise, etc. (e.g., Martin et al., 1997, 1999; Martin and Stapells, 2005; Whiting et al., 1998). For example, Billings et al. (2009) generated two tone levels (60- and 75-dB SPL) and six SNRs (quiet, 20, 10, 0, −10) by varying the levels of continuous white noise (Billings et al., 2009). As the SNR increased from −10 to +20, AEP amplitudes increased (0–100%) and the latencies shortened (70–80 ms shifts) in both N2 and P3 (Billings et al., 2009). In terms of areas of activation, when speech is presented in noise (or other types of degradation), increased right hemisphere activation (auditory cortices) has been demonstrated (Shtyrov et al., 1999), reflecting potential recruitment of additional brain regions for processing speech in noise. How this factor modulates processing of aspiration and of non-native phonetic cues is unknown.

### Gaps in literature, need for the present study, and aims

Most studies examining P1, N1, P2, N2, have used synthetic stimuli and a continuum of the target cue (e.g., −10 to 60 ms in 7 steps for a VOT continuum). Although this design is useful to study the nature of speech categories, this approach lacks ecological validity, given the unnaturalness of these speech stimuli. In addition, most cross-linguistic studies have examined non-native speech processing in only one direction. That is, these studies have not included native and non-native listeners, as well as a native and non-native contrast for both groups of listeners. Second, most cross-linguistic studies have presented speech stimuli in quiet condition. The ASP models predicts that non-native listeners and L2 learners will be more challenged in speech processing under noise conditions (Strange and Shafer, 2008; Shafer, 2024). Research has shown that native versus non-native differences in speech perception are more evident in noise conditions (e.g., Mattys et al., 2012; Mayo et al., 1997; Meador et al., 2000). Hence, it is important to examine whether P1, N1, P2, N2 differences between native and non-native listeners are influenced by noise.

Third, there is little work focusing on Tamil (compared to English or Hindi) speech sounds. Adding a Tamil group, for whom VOT is not contrastive will serve as a strong test of whether AEPs are affective by native language experience. This is the first study to examine the processing of the phonetic cues of aspiration versus voicing, and comparing these three language groups in a condition of noise masking versus in quiet. This study is part of a larger project. The behavioral findings from the study (Antony et al., 2024) show that speech perception is more difficult for non-native speech sounds relative to native speech sounds, specifically in noise, and for the aspirated speech sounds, even when the phonetic cues are somewhat similar across languages. The aim here is to examine neurophysiological processing using AEPs to examine whether there is a disassociation between the behavioral and electrophysiological findings. In addition, we address whether there is an interaction between linguistic experience versus condition (quiet versus noise) and versus acoustic feature (voicing versus aspiration).

Lastly, to our knowledge, no studies have examined AEPs to both native and non-native contrasts in a study crossing the nativeness of the stimuli to three groups. An interesting question is whether listeners show greater precision of encoding native- compared to non-native speech sounds. The objective for this study was to determine the neural encoding of Hindi and American English voiced and aspirated speech sounds, in Hindi, American English, and Tamil listeners with normal hearing, in quiet versus in noise. The study proposed here is novel because of the inclusion of aspiration, the language groups, inclusion of noise masking, and inclusion of auditory evoked potentials.

### Hypotheses

We hypothesize that AEPs in quiet reflect acoustic-phonetic, rather than phonological processing of the speech sounds because previous research has not observed language-specific group differences for early potentials (Chen and Small, 2015; Elangovan and Stuart, 2011; Sharma et al., 2000; Sharma and Dorman, 2000). In terms of condition, AEP responses were hypothesized to be less robust in all three groups with more resistance to the effects of the noise in the native listeners, predicted by the ASP model (Strange and Shafer, 2008). In terms of effects on stimuli, larger P1, N1, P2 amplitudes and prolonged latencies were predicted in response to aspirated stops when compared to unaspirated stops (Han et al., 2013). The AEP latencies of the long-lag aspirated stimuli [p^h^a] (i.e., Hindi phoneme /p^h^a/ and English phoneme /pa/) were hypothesized to be longer relative to the short-lag [pa] (Hindi phoneme /pa/ and English phoneme /ba/) (Sharma et al., 2000). Voiced stimuli (/ba/) as a reflection of the longer VOT (Sharma and Dorman, 2000; Elangovan and Stuart, 2011; Small and Werker, 2012). For the interactions, larger responses and shorter latencies were hypothesized for native listeners in quiet condition, specifically for voiced stimulus, relative to non-native listeners in noise condition and voiceless stimuli.

## Method

### Participants

Sixteen participants ages 20–45 years participated in each language group (Hindi, American English, Tamil). The number of participants was estimated using power analysis on pilot data (Antony and Martin, 2016) with apriori power set to 80%, effect size to 0.5 and an alpha level of 0.01 (N = 10, mean difference for P2 latency = 43.21 ms, sd = 17.22). The mean age of Hindi participants (10 female, 6 male) was 26.44 years (sd = 1.20), English participants (10 male, 6 female) was 29.38 years (sd = 1.72), and Tamil participants (8 male, 8 female) was 28.75 years (sd = 1.52). The participants were the same for both the behavioral study (Antony et al., 2024) and the electrophysiological study (this study).

Language proficiency was documented using the Language Experience and Proficiency Questionnaire (LEAP-Q) to document each participant’s native language, second language, and third language, if any (Marian et al., 2007). Table 1 displays the linguistic profiles of the three language groups.

**Table 1:** Linguistic profile of the participants.

Groups	Specific areas	L1		L2	
		Mean	SD	Mean	SD
Hindi participants	Speaking	9.31	0.87	9.19	0.83
	Understanding	9.38	0.72	9.13	0.89
	Reading	7.88	1.75	9.50	0.82
American English participants	Speaking	9.94	0.25	6.40	0.55
	Understanding	9.88	0.34	6.80	1.48
	Reading	9.88	0.34	7.20	0.84
Tamil participants	Speaking	9.94	0.25	9.22	1.08
	Understanding	10.00	0.00	9.44	0.89
	Reading	7.03	3.40	9.63	0.81

All Hindi and Tamil participants were L2 speakers of English. L2 was Spanish in three of them.

The participants had normal hearing sensitivity (pure tone thresholds of ≤25 dB HL for frequencies from 250–8,000 Hz, ANSI 1996) and middle ear function (admittance curve with a single peak between ± 50 daPa using a 226 Hz probe tone and present ipsilateral reflexes at 1000 Hz. Individuals reporting a history of recurrent middle ear problems, neurological problems, or language or learning problems were excluded. Prior to testing, the purpose of the study was explained to each participant and informed consent was obtained.

### Stimuli

**Figure 1:**
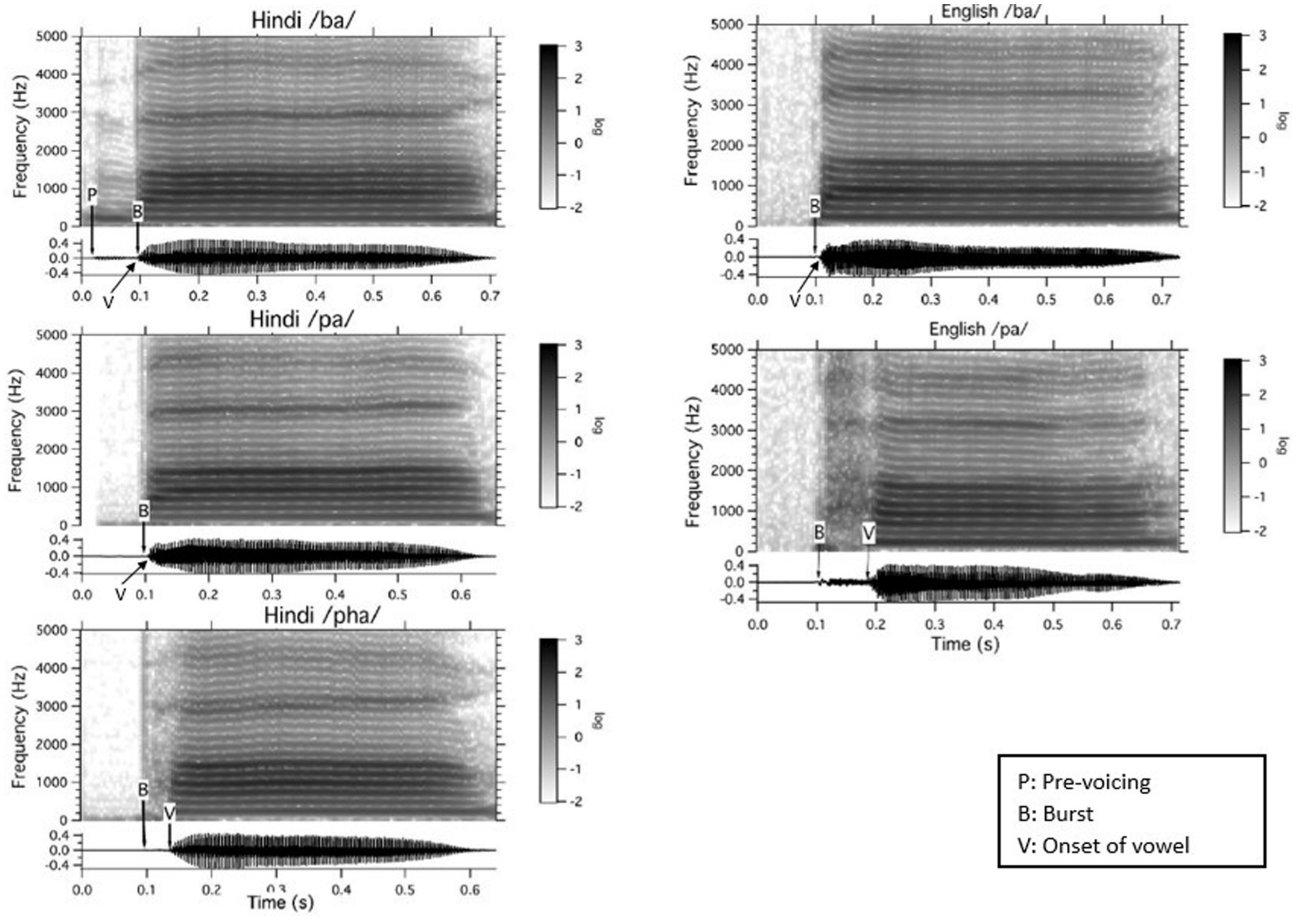
Waveforms and spectrograms of the five stimuli: Hindi /ba/, /pa/, /p^h^a/, and English /ba/, /pa/.

Natural digitized bilabial speech stimuli including Hindi /ba/, /pa/, and /p^h^a/ and English /ba/ and /pa/ were recorded using native Hindi and English female speakers, respectively. The CV stimuli used in this study is the same as those that were generated for the behavioral study (Antony et al., 2024). The talker for the Hindi stimuli, /ba/, /pa/, and /p^h^a/, was a 28-year-old female native Hindi speaker and, for the American English stimuli /ba/ and /pa/, the speaker was a 29-year-old native American English female. The stimuli were saved as mono recordings with a sampling rate of 44,100 Hz. The onset of the burst was aligned at 93 ms for each stimulus using padding (i.e., by inserting silence at the onset of stimuli). This was done for better interpretation of the ERP responses to onset responses and to allow comparison of responses across the stimuli. The Hindi /ba/ stimulus had prevoicing and was used to compute the duration of padding (silence) needed to align the burst of the other stimuli with the burst of the Hindi /ba/. Hence, 0 ms was the start of prevoicing in Hindi /ba/ and was the start of padding for the other four stimuli. Figure 1 displays waveforms and spectrograms of the five stimuli: Hindi /ba/, /pa/, /p^h^a/ and English /ba/, /pa/. Phonemic representation is denoted with ‘/ /’, whereas the phonetic representation is denoted with ‘[]’. Table 2 shows the acoustic characteristics of the five stimuli.

**Table 2:** Acoustic characteristics of the five stimuli.

Stimulus	Total duration without padding (ms)	Duration of consonant without padding (ms)	Duration of vowel (ms)	Total duration with padding (ms)	Duration of prevoicing (ms)	Duration of aspiration (ms)	VOT (ms)	Onset of vowel (ms)
Hindi stimuli								
/ba/ [ba]	709	98	611	709	86	0	−90	98
/pa/ [pa]	628	77	550	655	–	11	10	104
/p^h^a/ [p^h^a]	630	146	485	641	–	46	45	139
American English stimuli								
/ba/ [ba]	761	113	648	761	–	0	19	113
/pa/ [p^h^a]	713	201	512	713	–	81	107	199

In this study, to disentangle the responses to consonants from the vowel segments, a vowel-only stimuli were created for each corresponding CV stimuli. To accomplish this, each CV stimulus was segmented to produce the corresponding V-only condition. Specifically, the vowel segment was identified using spectrogram and the preceding consonant segment was set to zero. The vowel maintained the same temporal relation to the sampling window as in the original CV. The equalized stimuli (−18 dB) were presented binaurally at 70 dB SPL using insert earphones in two conditions - in quiet and in background noise (SNR 0). To create an ecologically valid noise, the envelope of a four-talker speech babble was extracted and filled with broadband noise (BBN). While the onset of the five stop consonants were aligned at 93 ms, the onset of noise was at 0 ms per trial; so, it was not played continuously. This alignment was done because when feedback was gathered from previous participants during pilot testing, they preferred some silence with noise being presented for each trial instead of noise being presented continuously.

### Procedures

The stimuli were presented using Gentask software and a Neuroscan 4.5 system. The listeners were tested in a double-walled sound attenuated booth and were instructed to watch a muted closed-captioned video of their choice. The stimuli were presented to the listeners at 70 dB SPL via EAR-3A insert earphones to both ears. Evoked potentials were recorded using a NeuroScan 4.5 system and a 32-channel cap. The electrode cap was placed on the participant’s head according to International 10–20 system, modified combinatorial nomenclature (Jasper, 1958; Sharbrough, 1991). An electrode placed at the nose served as reference and the electrode between FPz and Fz served as ground. Eyeblinks and eye-movements were monitored using an electrode below the left eye. Electrode impedances were maintained below 5 kOhms. Breaks were provided as and when required. During data acquisition, the EEG was digitized at a sampling rate of 1,000 Hz, amplified (1,000 times) and band-pass filtered (0.1–100 Hz with a roll-off of 6 dB/octave).

Two hundred and seventy-five repetitions of each stimulus were presented in separate runs. Quiet and noise conditions were randomly presented as separate runs. The order of presentation of the two languages was counterbalanced across listeners. Within each language, the order of presentation of the stimuli (CV and V-only) and the conditions were randomized. The onset-to-onset interval was 1,300 ms. This interval was selected because the longest stimulus is 761 ms and the latency window for the AEP components of interest including P1, N1, P2 is 75–250 ms. At least 500 ms of offset-to-onset time is preferred to avoid any overlap in responses to two sequential stimuli, even if the responses are delayed for some listeners. The estimated duration of the test was 3.5 h 1,300 ms * 275 trials * 10 stimuli * 2 conditions = 7,150,000 ms = 119 min ~ 2 h + 1 h (preparation time to place cap, gel, breaks, and instruction).

#### Data processing

Epochs of 1,000 ms were extracted from the continuous EEG which included a 100 ms pre-stimulus interval (i.e., −100 ms). So, time 0 ms was the onset of the stimulus presentation. Data cleaning was carried out offline. An eyeblink reduction algorithm was first applied to the EEG (Semlitsch et al., 1986). The data was processed offline by applying baseline correction to the pre-stimulus interval (−100 ms), and digital filtering (finite impulse response (FIR) filter, 1 to 30 Hz, 12 dB/octave, zero phase shift). Artifact rejection at +/−100 microvolts was applied to each epoch.

Averaged waveforms were computed for each participant, stimulus, and condition along with grand mean waveforms for each group. In addition, for each stimulus the averaged response to the V-only stimulus was subtracted from the response to the CV stimulus to isolate the response to consonant (C) in the CV condition.

The mean global field power (MGFP) from the grand mean waveforms was used to summarize the data. The largest positive and negative peak in the AEP were seen as positive peaks in the MGFP. The first step was to identify the peaks of interest from the grand mean waveforms for each stimulus in each condition using the following strategy: The peak amplitudes and peak latencies of onset P1, N1, P2, and N2 were measured at FCz, where the responses are typically largest to auditory stimuli, for each stimulus and condition (Bruneau et al., 1997). When additional peaks were present, selection of the peak was constrained by inspection of the pattern across all participants. The topography of the response was also used to select peaks because the AEP show inversion of responses seen at vertex in inferior posterior regions.

Time windows were then used to identify the peaks in the data from individual participants. For setting the time windows, the peak latencies were identified on the grand mean average waveforms. Then, 10 % of that value was used to formulate the time window. For example, if the latency of P1 for Hindi /pa/ in quiet was 163 ms, then the time window in this case was set to 147–179 ms (163–16 and 163 + 16). Peak latencies were measured at FCz, rather than using the GFP because the separate peaks were not always clearly delineated in the GFP. Further, in the subtracted waveforms (CV-V), the amplitudes were small and often too close to the noise floor. After identifying the latency of each peak, the GFP amplitude at that latency was selected for each participant. The latency and amplitude measures for the responses were analyzed. In this study, AEP data at the FCz electrode site will be measured because it is a midline-central electrode with relatively larger peak amplitudes. In the next publication, the data from temporal sites will be presented.

#### Statistical analysis

Mean and standard deviations of peak latencies and amplitudes at FCz were computed for each peak component on the average referenced waveforms (both responses to CV and CV-V, the subtracted waveforms), across participants as a function of language group, stimulus, and condition.

The Hindi and English stimuli were analyzed separately. Statistical analyses included mixed model ANOVA. The comparisons for the Hindi stimuli included group (Hindi, American English, Tamil), stimulus (/ba/, /pa/, /p^h^a/), and condition (quiet, noise) as factors, and for the English stimuli group (Hindi, American, Tamil), stimulus (/ba/, /pa/) and condition (quiet, noise). Results were considered significant when *p* < 0.05. Post-Hoc Fisher’s LSD analyses were done on the statistically significant findings. Results were considered significant when *p* < 0.05.

## Results

There was no significant difference in the ages across the three language groups [*F*(2, 45) = 1.072, *p* = 0.350]. Further, the data from LEAP-Q questionnaire was examined for differences across groups in L1 using mixed analysis of variance and no significant difference was present in their L1 proficiency [*F*(40, 18) = 2.0075, *p* = 0.056].

### Mean global field power (MGFP): morphology of waveforms

**Figure 2:**
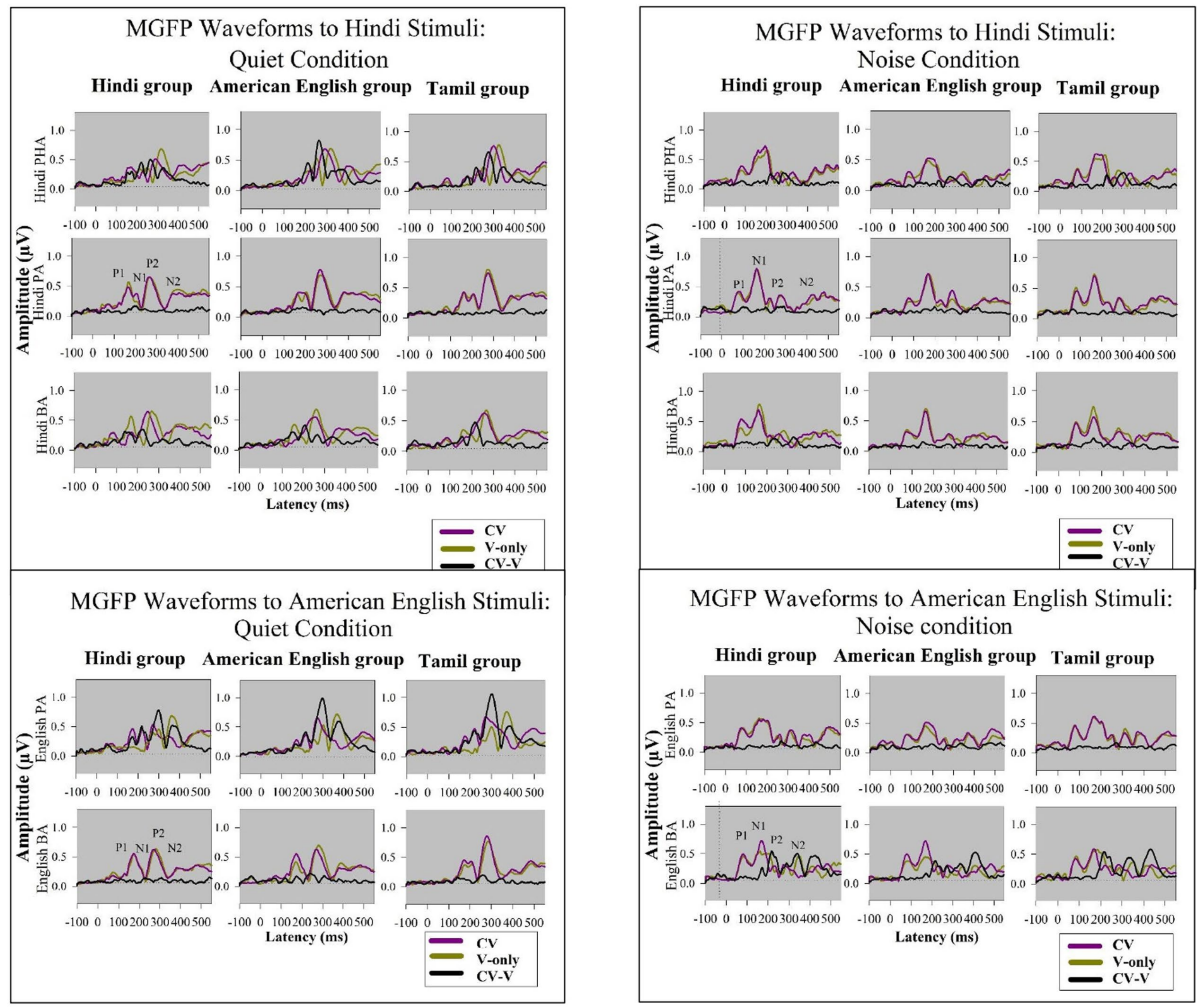
Mean global field power waveforms for the Hindi and American English stimuli in CV, V-only, and CV-V waveforms across all three language groups, in quiet and in noise.

MGFP waveforms were used in this study to summarize the results across all of the electrodes, to identify time windows and peaks of interest, and to understand its relevance to the stimuli. Figure 2 displays the mean global field power waveforms for the Hindi and American English stimuli in CV, V-only, and CV-V waveforms across all three language groups, in quiet and in noise.

The morphology of MGFP waveforms appeared to be similar across language groups. In quiet, P1, N1, P2, N2, components were identified. In noise, P1, N1, P2, N2 were identified as the peaks following the initial peak that corresponded to onset of vowel. The amplitudes in the CV-V responses were smaller relative to CV, and V-only responses, except for Hindi /p^h^a/ in quiet, American English /pa/ in quiet, and for American English /ba/ in noise. Further, the CV and V-only responses were so similar that they overlapped for most stimuli, except for Hindi /ba/, Hindi /p^h^a/, and American English /pa/ in quiet. The morphology of responses to Hindi /pa/ was similar to the morphology of responses to English /ba/. Also, for these two stimuli, the morphology of CV responses was similar to V-only responses. The CV responses and V-only responses to Hindi /p^h^a/ and to English /pa/, both which are aspirated, were noticeably different. The responses to vowel had longer latencies relative to CV responses.

In noise, however, these differences were not evident; the responses were smaller; and additional peaks were present in the N1 region. There was an early additional peak around 70 ms corresponding to onset of noise. Further, the latencies were longer and the responses to CV and V-only were more similar to each other, relative to responses in quiet. The only exception to this were the CV and V-only responses to English /ba/ in which both these were quite different. Lastly, P2 peak amplitudes were larger in quiet than in noise; P1 peak amplitudes were larger in noise than in quiet.

**Figure 3:**
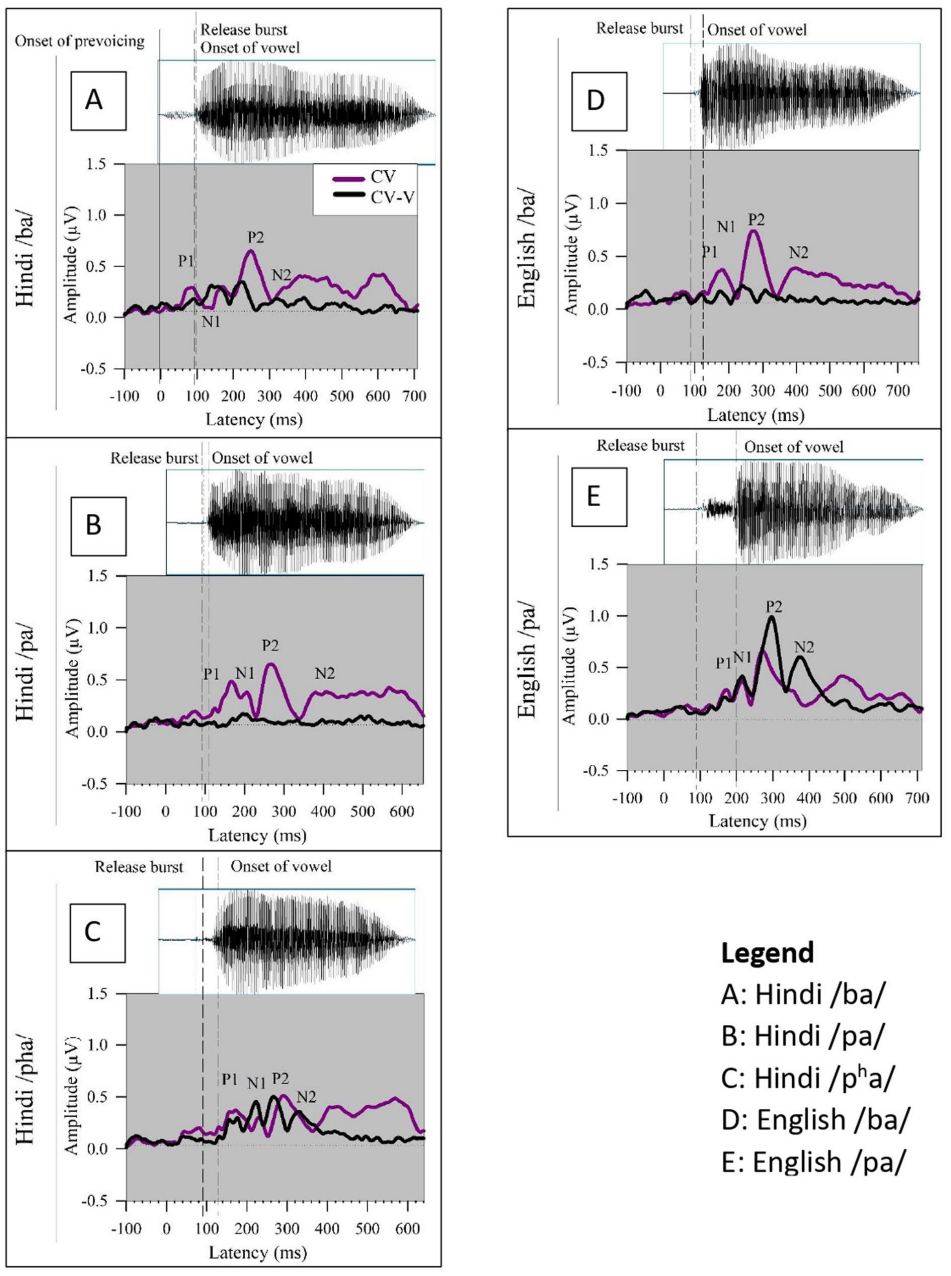
Relationship between the stimuli and the morphology of the MGFP waveforms.

Before the AEP amplitudes are measured, it is important to understand how the waveforms relate to the stimuli. Figure 3 illustrates the relationship between the stimuli, the MGFP waveforms, and the AEP components for each stimulus. The top panel include the waveforms of the stimulus and the bottom panel include the MGFP waveforms. The figure displays the MGFP of the CV waveforms in purple and the MGFP of the subtracted waveforms in black on bottom panel, in response to Hindi and English stimuli. The latency for P1 is normally approximately 50–75 ms post stimulus onset (Geisler et al., 1958; Hyde, 1997; Picton, 2013). In this study, the release burst for the stop consonants were aligned at 93 ms. Therefore, the P1 latency in response to the release burst would be predicted to be approximately 140–180 ms.

**Figure 4:**
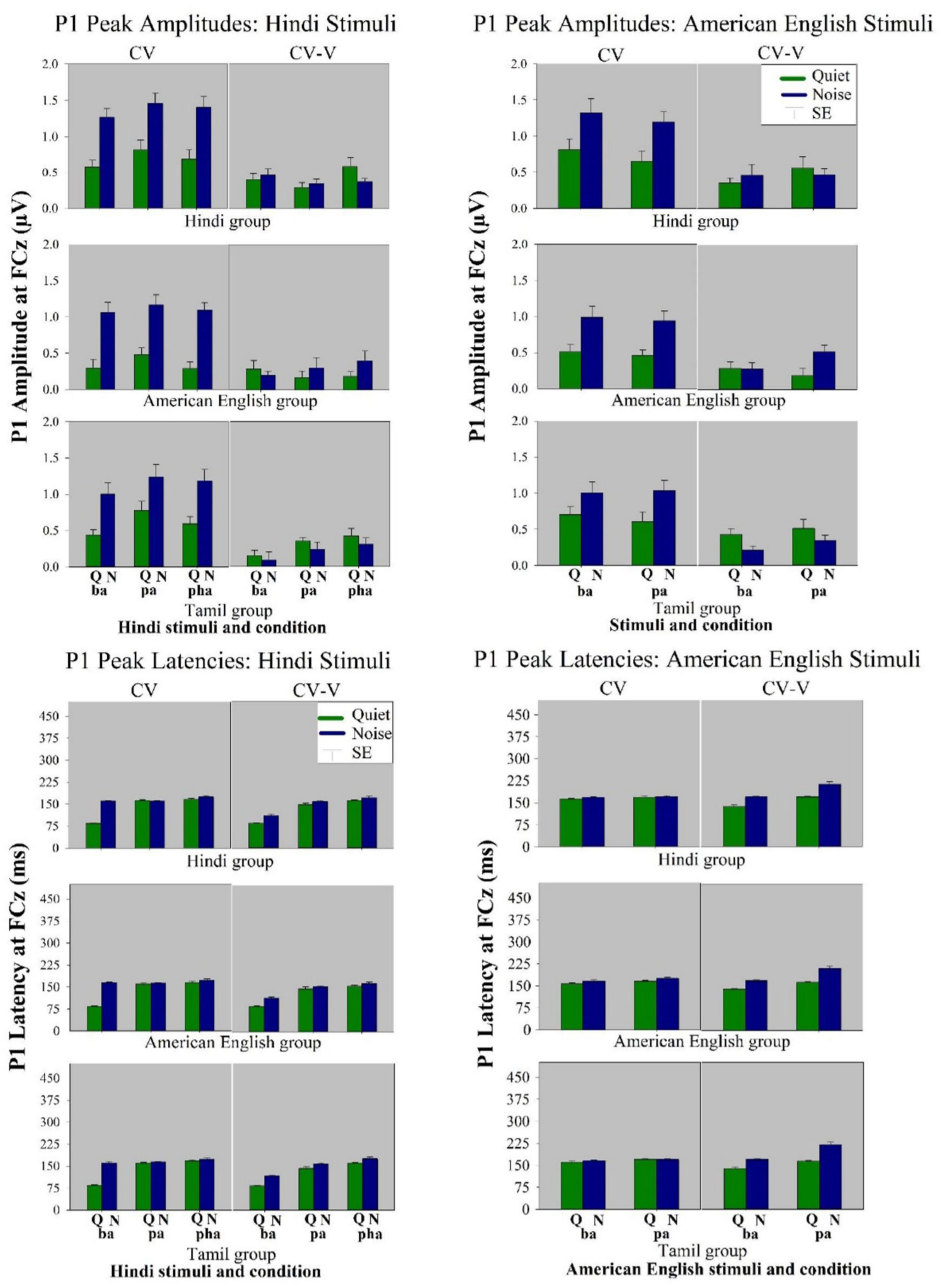
P1 amplitudes and latencies at FCz for the Hindi and English stimuli (CV, and CV-V) across all three language groups, in quiet and in noise.

From Figure 3, it appeared that the P1 was related to prevoicing, since it occurred well before this (see Figure 4). The N1 to Hindi /ba/ appears to correspond to prevoicing and onset of vowel, hence the complexity in response. For Hindi /pa/, P1 and N1 are probably related to both the release burst and vowel onset, which differ by less than 10 ms. For Hindi /p^h^a/, P1 was related to the release burst and onset of aspiration because the latency for P1 (i.e., 153–186 ms) corresponded to what would be expected in relation to the timing of the release burst (i.e., 163 ms) and aspiration (i.e., 170 ms). The P2 on CV waveforms was related to aspiration, because the latency for the peak (e.g., 263–321 ms) was later relative to what would be expected in response to release burst (i.e., 273 ms), but was too early to be associated with the onset of vowel (i.e., 319 ms). Also, the subtracted waveforms in responses to Hindi /p^h^a/ had larger amplitudes and distinct peaks relative to Hindi /pa/, possibly because given the complexity of the stimulus /p^h^a/; thus, the subtracted waveform was helpful in disentangling the responses to the consonant from the CV syllable. For the English /ba/, Figure 3 showed that P1 was associated with the burst and vowel onset, similar to Hindi /pa/, which is phonetically similar. For English /pa/, the peak latencies of P1 (i.e., 142–186 ms) and N1 (i.e., 183–238 ms) were associated with what would be expected in response to the release burst and aspiration, similar to Hindi /p^h^a/. Further, on the subtracted response, the increased P2 amplitude and timing also corresponded to aspiration.

AEP results at the FCz electrode site were presented in this study because it is a midline-central electrode with relatively large peak amplitudes. Table 3 shows the latency ranges for the five stimuli. Further, it reflects predominantly the primary auditory contributions to the responses. As indicated in the analyses section, results from the scalp distribution will be evaluated in a separate paper.

**Table 3:** Peak latency ranges for P1, N1, P2, N2 across stimuli.

	Stimulus	P1 (ms)	N1 (ms)	P2 (ms)	N2 (ms)
Hindi	Quiet condition				
/ba/	CV	76–94	121–147	220–270	333–407
	CV-V	77–95	121–140	201–270	333–407
/pa/	CV	76–121	121–229	239–293	346–424
	CV-V	77–172	121–229	230–280	275–430
/p^h^a/	CV	153–187	207–253	263–321	376–460
	CV-V	141–173	197–241	237–289	296–362
Hindi	Noise condition				
/ba/	CV	147–179	193–237	240–306	326–398
	CV-V	94–135	145–197	215–263	337–413
/pa/	CV	147–179	202–248	258–308	427–523
	CV-V	135–185	186–250	256–281	282–400
/p^h^a/	CV	144–220	231–283	294–360	392–480
	CV-V	135–209	201–245	258–308	344–420
English	Quiet condition				
/ba/	CV	142–174	183–223	241–300	325–424
	CV-V	107–165	166–204	205–280	281–400
/pa/	CV	142–186	183–239	243–289	336–550
	CV-V	107–183	166–240	205–325	281–414
English	Noise condition				
/ba/	CV	148–190	195–248	250–335	350–490
	CV-V	154–188	194–238	311–371	375–471
/pa/	CV	157–193	290–340	343–409	436–534
	CV-V	166–260	261–300	301–400	401–523

### P1 peak amplitudes and latencies at FCz

Figure 4 displays the grand mean P1 amplitudes and latencies at FCz for the Hindi and English stimuli (CV, and CV-V) across all three language groups, in quiet and in noise. Table 4 displays the statistical findings of the grand mean P1 peak amplitudes and latencies for CV and CV-V waveforms at FCz.

**Table 4:** Statistical findings of the P1 peak amplitudes and latencies at FCz.

			Hindi stimuli						English stimuli						
			Peak amplitudes			Peak latencies				Peak amplitudes			Peak latencies		
		Degree. of freedom	MS	*F*	*p*	MS	*F*	*p*	Degree. of freedom	MS	*F*	*p*	MS	*F*	*p*
CV	Group (G)	2	2.268	8.423	0.000	3.969	0.029	0.972	2	1.164	3.666	0.028	40.000	0.280	0.754	Condition (C)	1	31.189	115.807	0.000	59340.125	426.603	0.000	1	9.968	31.396	0.000	1074.000	7.600	0.006	Stimulus (S)	2	1.105	4.104	0.018	60435.823	434.480	0.000	1	0.280	0.883	0.349	1989.000	14.090	0.000	G*C	2	0.277	1.029	0.359	68.260	0.491	0.613	2	0.107	0.338	0.714	205.000	1.450	0.238	G*S	4	0.065	0.243	0.914	44.589	0.321	0.864	2	0.062	0.194	0.824	61.000	0.430	0.650	C*S	2	0.071	0.262	0.769	44811.885	322.158	0.000	1	0.039	0.123	0.726	91.000	0.640	0.424	G*C*S	4	0.003	0.009	0.999	54.224	0.390	0.816	2	0.015	0.047	0.954	38.000	0.270	0.764	Error	270	0.269			139.099			180	0.318			141.000		
	CV-V	Group (G)	2	0.669	4.723	0.010	220.045	0.777	0.461	2	0.300	1.815	0.166	27.000	0.060	0.939	Condition (C)	1	0.016	0.114	0.736	20016.670	70.663	0.000	1	0.003	0.019	0.890	75644.000	176.940	0.000	Stimulus (S)	2	0.370	2.612	0.075	114100.566	402.799	0.000	1	0.432	2.615	0.108	63329.000	148.140	0.000	G*C	2	0.212	1.495	0.226	327.253	1.155	0.317	2	0.499	3.021	0.051	168.000	0.390	0.676	G*S	4	0.202	1.427	0.225	319.670	1.129	0.343	2	0.007	0.040	0.961	158.000	0.370	0.691	C*S	2	0.027	0.193	0.825	2571.795	9.079	0.000	1	0.050	0.303	0.582	4116.000	9.630	0.002	G*C*S	4	0.197	1.390	0.237	30.910	0.109	0.979	2	0.280	1.693	0.187	194.000	0.450	0.637	Error	270	0.142			283.269			180	0.165			428.000		

Numbers in red represent significant findings (*p* < 0.05).

#### Effects of group

The P1 peak amplitudes were significantly larger for Hindi CV stimuli in Hindi participants (mean = 1.036 μV, s.e. = 0.128) relative to American English participants (mean = 0.728 μV, s.e. = 0.119) and Tamil participants (mean = 0.870 μV, s.e. = 0.133). A significant main effect of group [*F*(2, 270) = 8.422, *p =* 0.000, partial eta-squared = 0.058] was obtained. Post-hoc testing supported these observations, showing that the amplitudes for Hindi participants were significantly larger relative to the non-native participants [Hindi: *p <* 0.05 for all comparisons; American English vs. Tamil: *p* = 0.060].

For the English CV stimuli, P1 amplitudes were the lowest in native American-English listeners (mean = 0.727 μV, s.e. = 0.116) relative to non-native listeners (Hindi group: mean = 0.995 μV, s.e. = 0.160; Tamil: mean = 0.836 μV, s.e. = 0.138). A significant main effect of group was observed [*F*(2, 180) = 3.6656, *p =* 0.028, partial eta-squared = 0.0391]. The post-hoc test results revealed that the P1 amplitudes in the Hindi group were significantly larger relative to the American English group [American English vs. Hindi: *p <* 0.05; American English vs. Tamil: *p =* 0.273; Hindi vs. Tamil: *p =* 0.112].

#### Effects of condition

For the Hindi stimuli, the CV P1 responses were larger in noise (mean = 1.207 μV, s.e. = 0.144) relative to quiet (mean = 0.549 μV, s.e. = 0.110). A significant main effect of condition [*F*(1, 270) = 115.807, *p =* 0.000, partial eta-squared = 0.300] was observed. In the CV waveforms, as predicted, the mean latencies were significantly longer [*F*(1, 270) = 426.60, *p =* 0.000, partial eta-squared = 0.612) in noise (mean = 166.270 ms, s.e. = 2.919) relative to in quiet (mean = 137.562 ms, s.e. = 2.591).

For the American English CV stimuli, the P1 amplitudes were larger in the noise condition (mean = 1.081 μV, se = 0.156) relative to quiet (mean = 0.625 μV, s.e. = 0.120). A significant main effect of condition [*F*(1, 180) = 31.3959, *p =* 0.000, partial eta-squared = 0.1485] was present. The P1 latency for CV stimuli was longer in noise (mean = 168.969 ms, s.e. = 2.962) relative to in quiet (mean = 164.240 ms, s.e. = 2.929). A significant main effect of condition [*F*(1, 180) = 7.600, *p =* 0.006, partial eta-squared = 0.040] was obtained.

#### Effects of stimulus

The P1 peak amplitudes were significantly smaller [*F*(2, 270) = 4.103*, p =* 0.017, partial eta-squared = 0.029] for Hindi /ba/ (mean = 0.772 μV, s.e. = 0.120) relative to Hindi /pa/ (mean = 0.987 μV, s.e. = 0.137) and Hindi /p^h^a/ (mean = 0.875 μV, s.e. = 0.123). This finding also reflected in the post-hoc results where responses to Hindi /ba/ were significantly smaller relative to Hindi /pa/ [/ba/ vs. /pa/: *p* < 0.05], but not to Hindi /p^h^a/ [/ba/ vs. /p^h^a/: *p* = 0.171; /pa/ vs. /p^h^a/: *p* = 0.136]. The mean P1 peak latencies were shorter for /ba/ (123.343 ms, s.e. = 1.964) relative to /pa/ (162.052 ms, s.e. = 2.341) and /p^h^a/ (170.354 ms, s.e. = 3.959). A significant main effect of stimulus [*F*(2, 270) = 434.480, *p =* 0.000, partial eta-squared = 0.762] was present. Post-hoc test results revealed that the P1 latencies were significantly shorter for Hindi /ba/, short for /pa/, and long for /p^h^a/ [*p <* 0.05 for all comparisons].

For the English stimuli, the latency was longer for English /pa/ (mean = 169.823 ms, s.e. = 2.969) relative to /ba/ (mean = 163.385 ms, s.e. = 2.922) and this finding was statistically significant [*F*(1, 180) = 14.090, *p =* 0.000, partial eta-squared = 0.072].

#### Interactions

No significant interaction was present for the P1 amplitudes for the CV stimuli. In terms of P1 latencies, for Hindi CV stimuli, the interaction of condition x stimulus was significant [*F*(2, 270) = 322.158*, p =* 0.000, partial eta-squared = 0.704]. Post-hoc LSD test results revealed that the responses to Hindi /ba/ in quiet was significantly shorter (mean = 84.08 ms, s.e. = 1.702) relative to other Hindi stimuli in quiet or in noise [*p <* 0.05 for all comparisons]. In the American English CV, there was no significant interaction in the P1 amplitudes. In terms of latency, there were no significant interactions of group, condition, or stimulus in the P1 amplitudes for American English CV stimuli.

### N1 peak amplitudes and latencies at FCz

**Figure 5:**
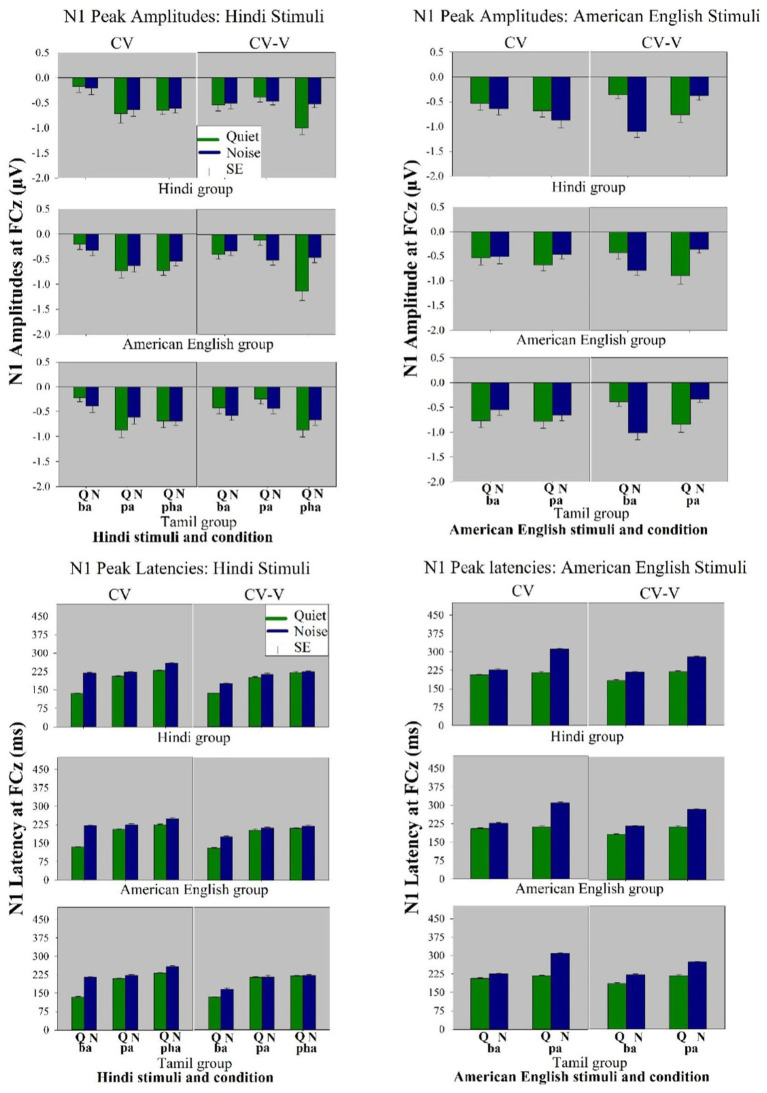
N1 amplitudes and latencies at FCz for the Hindi and English stimuli (CV, and CV-V) across all three language groups, in quiet and in noise.

Figure 5 displays the N1 amplitudes and latencies at FCz for the Hindi and English stimuli in CV, and CV-V waveforms across all three language groups, in quiet and in noise. Table 5 displays the statistical findings of the N1 peak amplitudes and latencies for CV and CV-V waveforms at FCz.

**Table 5:** Statistical findings of the N1 peak amplitudes and latencies at FCz.

			Hindi stimuli						English stimuli						
			Peak amplitudes			Peak latencies				Peak amplitudes			Peak latencies		
		Degree. of freedom	MS	*F*	*p*	MS	*F*	*p*	Degree. of freedom	MS	*F*	*p*	MS	*F*	*p*
CV	Group (G)	2	0.162	0.702	0.497	40.253	0.264	0.768	2	0.389	1.378	0.255	56.000	0.390	0.680	Condition (C)	1	0.111	0.480	0.489	128989.670	847.500	0.000	1	0.111	0.393	0.532	158126.000	1085.180	0.000	Stimulus (S)	2	5.745	24.893	0.000	104770.045	688.370	0.000	1	0.462	1.638	0.202	103324.000	709.080	0.000	G*C	2	0.006	0.027	0.973	81.983	0.539	0.584	2	0.468	1.659	0.193	135.000	0.920	0.399	G*S	4	0.015	0.064	0.992	270.191	1.775	0.134	2	0.104	0.368	0.693	11.000	0.080	0.924	C*S	2	0.412	1.786	0.170	30947.420	203.334	0.000	1	0.000	0.000	0.994	66083.000	453.510	0.000	G*C*S	4	0.093	0.403	0.807	39.545	0.260	0.903	2	0.104	0.369	0.692	31.000	0.220	0.807	Error	270	0.231			152.200			180	0.282			146.000		
	CV-V	Group (G)	2	0.172	0.839	0.433	16.885	0.075	0.927	2	0.024	0.108	0.898	14.000	0.080	0.927	Condition (C)	1	0.367	1.786	0.183	20842.014	92.941	0.000	1	0.118	0.531	0.467	112182.000	632.560	0.000	Stimulus (S)	2	4.444	21.629	0.000	123892.125	552.476	0.000	1	0.359	1.614	0.206	105703.000	596.030	0.000	G*C	2	0.247	1.200	0.303	505.024	2.252	0.107	2	0.287	1.290	0.278	252.000	1.420	0.244	G*S	4	0.102	0.495	0.740	480.807	2.144	0.076	2	0.141	0.633	0.532	247.000	1.390	0.251	C*S	2	2.861	13.924	0.000	8336.847	37.177	0.000	1	13.296	59.794	0.000	9282.000	52.340	0.000	G*C*S	4	0.266	1.293	0.273	77.811	0.347	0.846	2	0.077	0.345	0.708	361.000	2.030	0.134	Error	270	0.205			224.249			180	0.222			177.000		

Numbers in red represent significant findings (*p* < 0.05).

#### Effects of group

The N1 peak amplitudes and latencies were similar in all language groups for both Hindi and English stimuli. For example, the N1 peak latencies to Hindi stimuli was similar across language groups: in Hindi participants (CV: mean = 211.510 ms, s.e. = 2.784; CV-V: mean = 194.635 ms, s.e. = 3.245), American English participants (CV: mean = 210.354 ms, s.e. = 3.131; CV-V: mean = 194.083 ms, s.e. = 3.648), and Tamil participants (CV: mean = 211.438 ms, s.e. = 3.102; CV-V: mean = 194.906 ms, s.e. = 3.645).

#### Effects of condition

The N1 latency for Hindi CV stimuli was longer in noise (mean = 232.263 ms, s.e. = 3.531) relative to quiet condition (mean = 189.937 ms, s.e. = 2.479). A main effect of condition was present [*F*(1, 270) = 847.50, *p =* 0.000, partial eta-squared = 0.758].

For the American English CV stimuli, the N1 latency was longer in the noise condition (mean = 268.250 ms, s.e. = 3.272) relative to in quiet (mean = 210.254 ms, s.e. = 2.704) and this finding was statistically significant [*F*(1, 180) = 1085.180, *p =* 0.000, partial eta-squared = 0.857].

#### Effects of stimulus

The amplitude for Hindi CV /ba/ (mean = −0.254 μV, s.e. = 0.110) was smaller relative to Hindi /pa/ (mean = −0.700 μV, s.e. = 0.142) and /p^h^a/ (mean = −0.651, s.e = 0.099). A significant main effect of stimulus was present [*F*(2, 270) = 24.893, *p =* 0.000, partial eta-squared = 0.155]. The *post-hoc* testing added more evidence to support this finding as /ba/ was significantly smaller relative to the other two stimuli [/ba/: *p* < 0.05 for all comparisons; /pa/ vs. /p^h^a/: *p* = 0.474]. The latencies for Hindi CV /p^h^a/ were longer (mean = 241.958 ms, s.e. = 3.250) relative to Hindi /ba/ (mean = 176.250 ms, s.e. = 2.894) and /pa/ (mean = 215.093 ms, s.e. = 2.871). A main effect of stimulus was present [*F*(2, 270) = 688.370, *p =* 0.000, partial eta-squared = 0.836]. The post-hoc testing revealed that the performance for all three stimuli were significantly different with significantly longer latencies for Hindi /p^h^a/ and shorter latencies for Hindi /ba/ [*p <* 0.05 for all comparisons].

For the American English CV stimuli, the N1 latency, as expected, was longer in response to English /pa/ (mean = 262.750 ms, s.e. = 3.104) relative to English /ba/ (mean = 216.354 ms, s.e. = 2.873). A significant main effect of stimulus [F(1, 180) = 709.080, *p =* 0.000, partial eta-squared = 0.797] was present.

#### Interactions

For Hindi CV stimuli, no significant interaction was observed for N1 peak amplitudes. In the CV-V subtracted waveforms, a significant interaction of condition x stimulus [F(2, 270) = 13.924, *p =* 0.000, partial eta-squared = 0.093] was observed. Further, on post-hoc analyses, the interactions were evident. The N1 amplitudes were significantly larger for Hindi /p^h^a/ in quiet (mean = −1.000 μV, s.e. = 0.065) and for smaller for Hindi /pa/ in quiet (mean = −0.250 μV, s.e. = 0.065) relative to other stimuli and condition [*p <* 0.05 for all /pa/ and /p^h^a/ comparisons; for all other comparisons: *p-range = 0.316 to 0.990*]. In terms of latency, a significant interaction of condition x stimulus for the CV Hindi stimuli was present [F(2, 270) = 203.334, *p =* 0.000, partial eta-squared = 0.600]. *Post hoc* testing revealed that the latencies for Hindi /ba/ was the shortest, short for Hindi /pa/ and long for Hindi /p^h^a/, both in quiet and in noise, but with longer latencies in noise and this finding was statistically significant [*p <* 0.05 for all comparisons]. For example, the latency for Hindi /ba/ in quiet was shortest (mean = 134.562 ms, s.e. = 1.780) and was longest for Hindi /p^h^a/ in noise (mean = 255.395 ms, s.e. = 1.780).

There was no significant interaction between group, condition, or stimulus on N1 peak amplitudes for the American English CV stimuli. There was also a significant interaction of condition x stimulus for N1 peak latencies in response to English CV stimuli [*F*(1, 180) = 453.510, *p =* 0.000, partial eta-squared = 0.715]. Post-hoc results [*p <* 0.05 for all comparisons] showed that the English /pa/ in noise had the longest N1 peak latency (mean = 310.00 ms, s.e. =1.742) and English /ba/ had the shortest latency (mean = 206.20 ms, s.e. = 1.742), but the differences in latency were greatest in noise.

### P2 peak amplitudes and latencies at FCz

**Figure 6:**
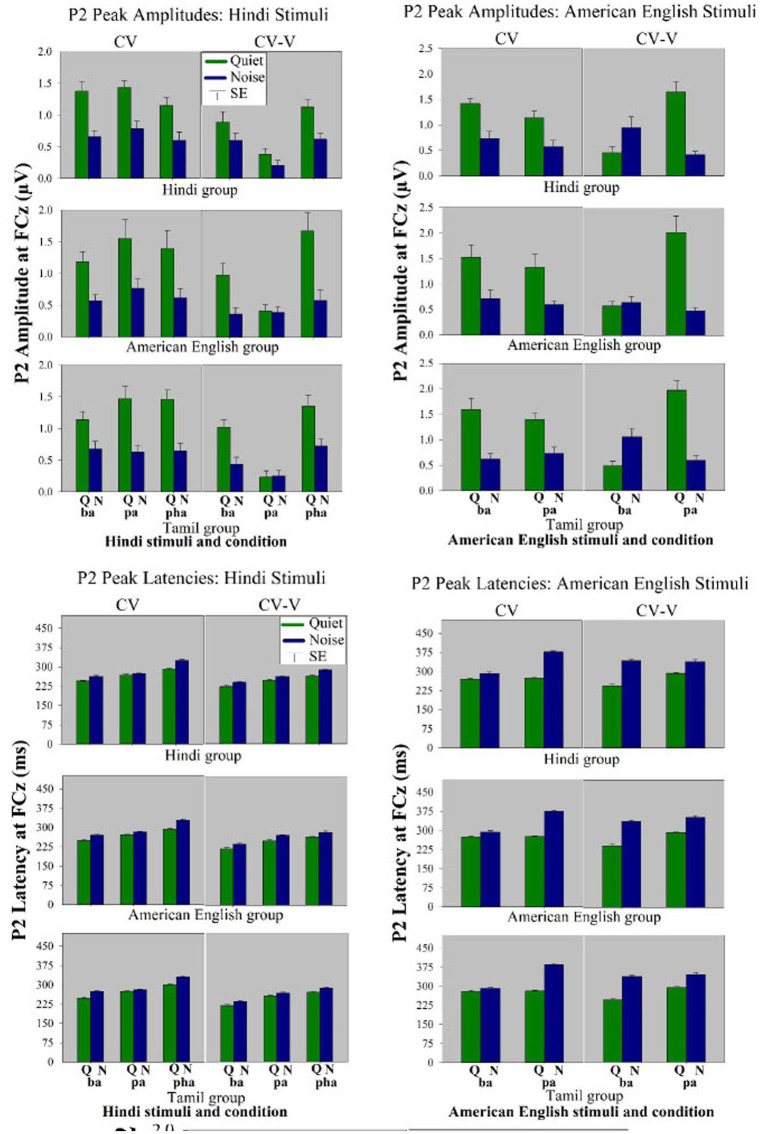
P2 amplitudes and latencies at FCz for the Hindi and English stimuli (CV, and CV-V) across all three language groups, in quiet and in noise.

Figure 6 shows the P2 amplitudes and latencies at FCz for Hindi and English stimuli in quiet and in noise. Table 6 displays the statistical findings of the P2 peak amplitudes and latencies for CV and CV-V waveforms at FCz.

**Table 6:** Statistical findings of the P2 peak amplitudes and latencies at FCz.

			Hindi stimuli						English stimuli						
			Peak amplitudes			Peak latencies				Peak amplitudes			Peak latencies		
		Degree. of freedom	MS	F	p	MS	F	p	Degree. of freedom	MS	F	p	MS	F	p
CV	Group (G)	2	0.003	0.008	0.992	999.003	4.897	0.008	2	0.219	0.536	0.586	689.000	2.570	0.079	Condition (C)	1	34.545	85.118	0.000	30834.722	151.143	0.000	1	26.357	64.540	0.000	169516.000	633.440	0.000	Stimulus (S)	2	0.762	1.878	0.155	71212.462	349.063	0.000	1	0.896	2.194	0.140	93678.000	350.050	0.000	G*C	2	0.053	0.131	0.878	73.670	0.361	0.697	2	0.167	0.409	0.665	122.000	0.460	0.635	G*S	4	0.270	0.665	0.617	40.665	0.199	0.939	2	0.118	0.289	0.749	102.000	0.380	0.685	C*S	2	0.167	0.412	0.663	3736.108	18.313	0.000	1	0.355	0.868	0.353	86488.000	323.180	0.000	G*C*S	4	0.164	0.403	0.807	133.509	0.654	0.624	2	0.063	0.155	0.857	177.000	0.660	0.518	Error	270	0.406			204.010			180	0.408			268.000		
	CV-V	Group (G)	2	0.257	0.850	0.428	108.233	0.416	0.660	2	0.432	1.073	0.344	97.000	0.190	0.829	Condition (C)	1	13.526	44.793	0.000	22419.031	86.198	0.000	1	12.174	30.222	0.000	261444.000	502.780	0.000	Stimulus (S)	2	11.834	39.191	0.000	56661.358	217.856	0.000	1	11.674	28.980	0.000	37325.000	71.780	0.000	G*C	2	0.409	1.354	0.259	178.823	0.688	0.504	2	0.662	1.643	0.196	212.000	0.410	0.666	G*S	4	0.278	0.922	0.451	372.488	1.432	0.224	2	0.378	0.939	0.393	444.000	0.850	0.428	C*S	2	2.900	9.604	0.000	51.260	0.197	0.821	1	37.096	92.092	0.000	22729.000	43.710	0.000	G*C*S	4	0.350	1.159	0.329	75.443	0.290	0.884	2	0.119	0.294	0.745	333.000	0.640	0.529	Error	270	0.302			260.086			180	0.403			520.000		

Numbers in red represent significant findings (*p* < 0.05).

#### Effects of group

For the Hindi stimuli, in the CV waveforms, the P2 amplitudes in Hindi participants (mean = 1.000 μV, s.e. = 0.119) were similar to the responses in non-native participants (American English participants: mean = 1.011 μV, s.e. = 1.191; Tamil: mean = 1.004 μV, s.e. = 0.136). However, from Figure 6, it can be noted that in the subtracted waveforms for Hindi stimuli, the P2 amplitudes were larger in Hindi participants (mean = 0.405 μV, s.e. = 0.081) relative to non-native participants (American English participants: mean = 0.261 μV, s.e. = 0.100; Tamil: mean = 0.260 μV, s.e. = 0.089). A significant main effect of group [*F*(2, 270) = 4.723, *p =* 0.010, partial eta-squared = 0.033] was present to CV-V stimuli. Post-hoc testing revealed significantly larger P2 amplitudes in the Hindi participants relative to the other two groups [Hindi: *p <* 0.05 for all comparisons, American English vs. Tamil: *p =* 0.990]. This is an important finding that reveals the effects of consonant because there was no significant effect of group for the CV stimulus analysis. The significant effect in the subtracted waveforms disentangles the effects of vowel from the CV.

#### Effects of condition

For the Hindi CV stimuli, the P2 amplitudes were larger in quiet (mean = 1.351 μV, s.e. = 0.175), relative to in noise (mean = 0.659 μV, s.e. = 0.123). A significant effect of condition [*F*(1, 270) = 85.118, *p =* 0.000, partial eta-squared = 0.239] was present. The P2 latency for Hindi CV stimuli was longer in noise (mean = 292.131 ms, s.e. = 3.712) relative to in quiet (mean = 271.437 ms, s.e. = 3.166). This finding was statistically significant [F(1, 270) = 151.143, *p =* 0.000, partial eta-squared = 0.358].

For the American English CV stimuli, the P2 amplitude in noise (mean = 0.659 μV, s.e. = 0.123) was smaller relative to in quiet (mean = 1.400 μV, s.e. = 0.177). A main effect of condition was present [*F*(1, 180) = 64.540, *p =* 0.000, partial eta-squared = 0.263]. The pattern of results was also similar in the subtracted waveforms with significantly larger P2 amplitudes [F(1, 180) = 30.222, *p =* 0.000, partial eta-squared = 0.143] in quiet (mean = 1.194 μV, s.e. = 0.164) relative to in noise (mean = 0.690 μV, s.e. = 0.118). The P2 latencies for the English CV stimuli were longer in noise (mean = 335.271 ms, s.e. = 4.865) relative to in quiet (mean = 275.844 ms, s.e. = 2.927) and this finding was statistically significant [F(1, 180) = 633.440, *p =* 0.000, partial eta-squared = 0.778].

#### Effects of stimulus

A significant main effect of stimulus was present for the Hindi CV stimuli [F(2, 270) = 349.063, *p =* 0.000, partial eta-squared = 0.721]. Post-hoc testing indicated that the P2 latencies were significantly longer [*p <* 0.05 for all comparisons] for Hindi /p^h^a/ (mean = 311.677 ms, s.e. = 3.650) relative to Hindi /ba/ (mean = 258.375 ms, s.e. = 3.998) and /pa/ (mean = 275.302 ms, s.e. = 2.669).

Similar to CV responses, the P2 latencies in the subtracted responses were significantly longer [F(2, 270) = 217.856, *p =* 0.000, partial eta-squared = 0.617] for Hindi /p^h^a/ (mean = 276.541 ms, s.e. = 3.320), relative to Hindi /pa/ (mean = 259.197 ms, s.e. = 3.569) and /ba/ (mean = 228.562 ms, s.e. = 4.776). Post-hoc testing revealed that the P2 latencies was significantly longer for Hindi /p^h^a/ and significantly shorter for Hindi /ba/ relative to Hindi /pa/ [*p <* 0.05 for all comparisons]. In terms of latency, as expected, the mean P2 latency was longer in response to English /pa/ (mean = 327.646 ms, s.e. = 3.751) relative to English /ba/ (mean = 283.469 ms, s.e. = 4.041). A main effect of stimulus was present [*F*(1, 180) = 350.050, *p =* 0.000, partial eta-squared = 0.660].

#### Interactions

In the Hindi CV responses, no significant interactions were present in terms of P2 amplitude. In terms of P2 latencies, a significant interaction of condition x stimulus was observed in response to the Hindi CV stimuli [*F*(2, 270) = 18.313*, p =* 0.000, partial eta-squared = 0.119]. The latencies were significantly short for /ba/, long for /pa/, and longest for Hindi /p^h^a/. Further, the latencies were significantly longer in noise relative to quiet, but less so to /pa/ than the other two stimuli. For example, the latency of Hindi /ba/ in quiet was shorter (mean = 247.208 ms, s.e. = 2.061), relative to in noise (mean = 269.541 ms, s.e. = 2.061). *Post hoc* LSD test results revealed significantly longer latencies [/p^h^a/ in noise and /ba/ in quiet: *p <* 0.05 for all comparisons] for Hindi /p^h^a/ in noise (mean = 327.812 ms, s.e. = 2.061) and significantly shorter for Hindi /ba/ in quiet (mean = 247.208 ms, s.e. = 2.061). For the American English CV stimuli, there were no significant interactions of group, condition, or stimulus in the P2 peak amplitudes.

In terms of latency, significant interactions of condition x stimulus were present [CV: *F*(1, 180) = 323.18, *p =* 0.000, partial eta-squared = 0.642]. Post-hoc testing revealed that the mean P2 latency was longer in response to English /pa/ (quiet: mean = 276.708 ms, s.e. = 2.361; noise: mean = 378.583 ms, s.e. = 2.361) relative to English /ba/ (quiet: mean = 274.979 ms, s.e. = 2.361; noise: mean = 291.958 ms, s.e. = 2.361), but the difference was greater in noise [/ba/ and /pa/ in noise: *p <* 0.05 for all comparisons, /ba/ in quiet vs. /pa/ in quiet: *p =* 0.605].

### N2 peak amplitudes and latencies at FCz

**Figure 7:**
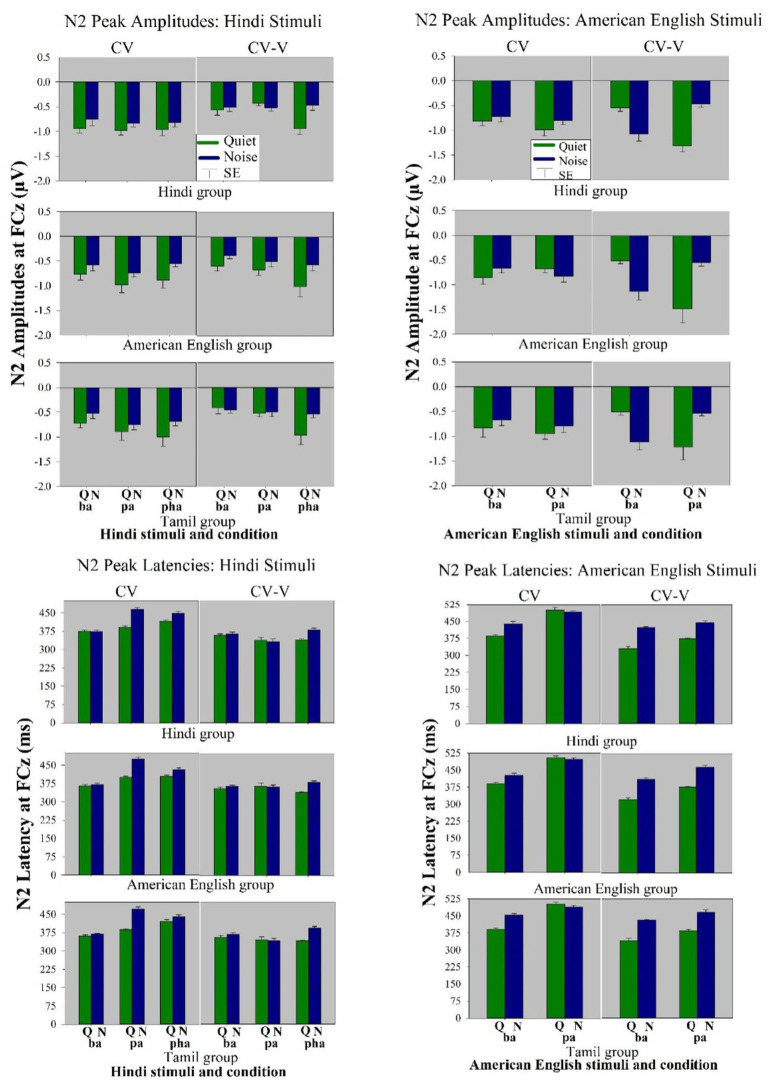
N_2_ amplitudes and latencies at FCz for the Hindi and English stimuli (CV, and CV-V) across all three language groups, in quiet and in noise.

Figure 7 displays the N2 amplitudes and latencies at FCz for the Hindi stimuli and English in CV, and CV-V waveforms across all three language groups, in quiet and in noise. Table 7 displays the statistical findings of the P2 peak amplitudes and latencies for CV and CV-V waveforms at FCz.

**Table 7:** Statistical findings of the N2 peak amplitudes and latencies at FCz.

			Hindi stimuli						English stimuli						
			Peak Amplitudes			Peak Latencies				Peak Amplitudes			Peak Latencies		
		Degree. of freedom	MS	*F*	*p*	MS	*F*	*p*	Degree. of freedom	MS	*F*	*p*	MS	*F*	*p*
CV	Group (G)	2	0.495	2.155	0.118	413.198	0.693	0.501	2	0.097	0.426	0.654	505.000	0.520	0.598
	Condition (C)	1	3.224	14.044	0.000	94395.125	158.353	0.000	1	0.547	2.391	0.124	19724.000	20.160	0.000
	Stimulus (S)	2	0.561	2.445	0.089	118406.167	198.632	0.000	1	0.314	1.374	0.243	333000.000	340.390	0.000
	G*C	2	0.054	0.235	0.790	30.010	0.050	0.951	2	0.086	0.377	0.686	555.000	0.570	0.568
	G*S	4	0.124	0.538	0.708	1460.833	2.451	0.046	2	0.087	0.379	0.685	1484.000	1.520	0.222
	C*S	2	0.060	0.263	0.769	33358.792	55.961	0.000	1	0.082	0.357	0.551	43863.000	44.840	0.000
	G*C*S	4	0.027	0.118	0.976	495.583	0.831	0.506	2	0.224	0.978	0.378	1192.000	1.220	0.298
	Error	270	0.230			596.107			180	0.229			978.000		
CV-V	Group (G)	2	0.068	0.353	0.703	2566.125	2.270	0.105	2	0.075	0.212	0.809	3389.000	3.710	0.026
	Condition (C)	1	2.498	13.006	0.000	18915.125	16.735	0.000	1	0.673	1.911	0.169	347565.000	380.740	0.000
	Stimulus (S)	2	1.919	9.995	0.000	6989.573	6.184	0.002	1	0.602	1.711	0.193	83625.000	91.610	0.000
	G*C	2	0.133	0.694	0.500	214.125	0.189	0.828	2	0.079	0.225	0.799	200.000	0.220	0.803
	G*S	4	0.083	0.432	0.785	2651.292	2.346	0.055	2	0.080	0.226	0.798	1908.000	2.090	0.127
	C*S	2	1.251	6.514	0.002	15777.635	13.959	0.000	1	23.717	67.361	0.000	1832.000	2.010	0.158
	G*C*S	4	0.064	0.333	0.856	100.729	0.089	0.986	2	0.073	0.208	0.812	473.000	0.520	0.597
	Error	270	0.192			1130.281			180	0.352			913.000		

Numbers in red represent significant findings (*p* < 0.05).

### Effects of group

For the Hindi stimuli, in the CV waveforms, the N2 amplitude were similar in all the participants (e.g., Hindi: mean = −0.879 μV, s.e. = 0.099) relative to non-native participants (American English participants: mean = −0.748 μV, s.e. = 0.118; Tamil participants: mean = −0.762 μV, s.e. = 0.125).

For the English stimuli, the N2 latencies in Hindi listeners (mean = 453.406 ms, s.e. = 7.964) were similar to English listeners (mean = 455.234 ms, s.e. = 7.657) and Tamil listeners (mean = 458.922 ms, s.e. = 7.049).

#### Effects of condition

In the CV responses to Hindi stimuli, N2 amplitudes were larger in quiet (mean = −0.902 μV, s.e. = 0.132), relative to in noise (mean = −0.691 μV, s.e. = 0.096). There was a significant main effect of condition [*F*(1, 270) = 14.044, *p =* 0.000, partial eta-squared = 0.049]. In terms of latency, longer N2 peak latency was observed in noise (mean = 427.270 ms, s.e. = 6.463) relative to quiet condition (mean = 391.062 ms, s.e. = 5.494). There was a main effect of condition [*F*(1, 270) = 158.35, *p =* 0.000, partial eta-squared = 0.369].

The N2 latency in response to English CV stimuli was longer in noise (mean = 465.990 ms, s.e. = 8.017) relative to in quiet (mean = 445.719 ms, s.e. = 7.096) and this finding was statistically significant [*F*(1, 180) = 20.160, *p =* 0.000, partial eta-squared = 0.100].

#### Effects of stimulus

In the subtracted waveforms, the N2 peak amplitudes for Hindi /ba/ (mean = −0.485 μV, s.e. = 0.090) and Hindi /pa/ (mean = −0.524 μV, s.e. = 0.081) were smaller relative to the N2 amplitude for Hindi /p^h^a/ (mean = −0.747 μV, s.e = 0.134). This finding was statistically significant [*F*(2, 270) = 9.9950, *p =* 0.000, partial eta-squared = 0.068]. Post-hoc LSD Fisher’s test revealed that the N2 amplitudes for Hindi /p^h^a/ was significantly larger [MS = 0.19203, df = 270.00, *p <* 0.05]. On the CV Hindi stimuli, the N2 latencies for Hindi /p^h^a/ (mean = 427.000 ms, s.e. = 6.328) were longer and the responses to Hindi /ba/ (mean = 368.708 ms, s.e. = 5.100) were shorter, relative to Hindi /pa/ (mean = 431.791 ms, s.e. = 6.508). A main effect of stimulus was present [F(2, 270) = 198.63, *p =* 0.000, partial eta-squared = 0.595]. Post-hoc testing showed that the N2 latencies in response to Hindi /ba/ were significantly shorter relative to /pa/ and p^h^a/ [/ba/: *p <* 0.05 for all comparisons, except /pa/ vs. /p^h^a/: *0.175*].

For the American English stimuli, as expected, the mean N2 latency was longer in response to English CV /pa/ (mean = 497.500 ms, s.e. = 7.436) relative to English /ba/ (mean = 414.208 ms, s.e. = 7.678). A main effect of stimulus was present [F(1, 180) = 340.39, *p =* 0.000, partial eta-squared = 0.654].

#### Interactions

There were no significant interactions of group, condition, or stimulus in the N2 peak amplitudes for the CV Hindi stimuli. In terms of N2 peak latencies, a significant interaction of group x stimulus was present [*F*(4, 270) = 2.451, *p = 0.046*, partial eta-squared = 0.035] in the CV waveforms. The latencies for Hindi /ba/ were significantly shorter relative to the latencies for Hindi /pa/ and Hindi /p^h^a/, in all three groups (Hindi group: mean = 374.00 ms, s.e = 4.316; English group: mean = 367.531 ms, s.e. = 4.316; Tamil group: mean = 364.593, s.e. = 4.316). For Hindi /pa/ versus Hindi /p^h^a/, the N2 latencies for both the stimuli were similar in Hindi listeners (/pa/: mean = 428.750 ms, s.e. = 4.316; /p^h^a/: 431.687, s.e. = 4.316) and in Tamil listeners (/pa/: mean = 429.718 ms, s.e. = 4.316; /p^h^a/: 431.343, s.e. = 4.316). However, the N2 peak latency in response to Hindi /pa/ was slightly longer relative to Hindi /p^h^a/ in American English listeners. (/pa/: mean = 436.906 ms, s.e. = 4.316; /p^h^a/: 417.968, s.e. = 4.316). This was an unexpected finding and was not hypothesized. However, the post-hoc results from Fisher’s LSD test supported this finding [*p <* 0.05].

Also, a significant condition x stimulus interaction was present for the N2 peak latencies in the CV stimuli [F(2, 270) = 55.961, *p =* 0.000, partial eta-squared = 0.293]. The N2 latencies in response to Hindi /ba/ were similar in quiet (mean = 366.250 ms, s.e = 3.524) and in noise (mean = 371.166 ms, s.e = 3.524). However, for Hindi /pa/ and Hindi /p^h^a/, the latencies were significantly longer in noise relative to in quiet. For example, the N2 peak latency for Hindi /p^h^a/ in quiet was 413.875 ms (s.e = 3.524), relative to in noise 440.125 ms (s.e = 3.524). The post-hoc results supported this finding [*p <* 0.05 for all comparisons, except /ba/ in quiet vs. in noise: *p* = 0.324].

For the American English CV stimuli, there was no significant interaction of group, condition, or stimulus in the N2 peak amplitudes. In terms of latency, there was a significant interaction of condition x stimulus in the CV waveforms [F(1, 180) = 44.840, *p =* 0.000, partial eta-squared = 0.199]. Post-hoc testing indicated that the N2 latencies for English /ba/ were significantly shorter in quiet and longer in noise [*p <* 0.05]. However, the N2 latencies for English /pa/ were similar in quiet versus in noise (quiet: mean = 439.458 ms, s.e = 4.514; noise: mean = 492.520 ms, s.e = 4.514; *p =* 0.120), This finding is in contrast to what was obtained in response to the Hindi stimuli. In the subtracted waveforms, there was no significant interaction between group, condition, or stimulus.

Tables 8, 9 is a summary of the findings showing the significant effects of group, condition, and stimuli for both amplitudes (Table 8) and latencies (Table 9). The two tables also highlight when effects of condition and stimuli were significant.

**Table 8:** Summary of statistical findings for P1, N1, P2, and N2 peak amplitudes at FCz.

AEP component	Language of stimuli	Context	Group	Condition	Stimulus	Interaction
P1	Hindi stimuli	CV	X	X	X	–
		CV-V	X	–	–	–
	American English stimuli	CV	X	X	–	–
		CV-V	–	–	–	–
N1	Hindi stimuli	CV	–	–	X	–
		CV-V	–	–	X	X
	American English stimuli	CV	–	–	–	–
		CV-V	–	–	–	X
P2	Hindi stimuli	CV	–	X	–	–
		CV-V	X	–	–	–
	American English stimuli	CV	–	X	–	–
		CV-V	–	X	X	X
N2	Hindi stimuli	CV	–	X	X	-
		CV-V	–	X	X	X
	American English stimuli	CV	–	–	–	–
		CV-V	–	–	–	X

“X” in the table represents the significant findings (*p* < 0.05) and “–” in the table represent the findings that were not statistically significant (*p* > 0.05).

**Table 9:** Summary of statistical findings for P1, N1, P2, and N2 peak latencies at FCz.

AEP component	Language of stimuli	Context	Group	Condition	Stimulus	Interaction
P1	Hindi stimuli	CV	–	X	X	X
		CV-V	–	X	X	X
	American English stimuli	CV	–	X	X	–
		CV-V	–	X	X	X
N1	Hindi stimuli	CV	–	X	X	X
		CV-V	–	X	X	X
	American English stimuli	CV	–	X	X	X
		CV-V	–	X	X	X
P2	Hindi stimuli	CV	X	X	X	X
		CV-V	–	X	X	–
	American English stimuli	CV	–	X	X	X
		CV-V	–	X	X	X
N2	Hindi stimuli	CV	–	X	X	X
		CV-V	–	X	X	X
	American English stimuli	CV	–	X	X	X
		CV-V	X	X	X	–

“X” in the table represents the significant findings (*p* < 0.05) and “–” in the table represent the findings that were not statistically significant (*p* > 0.05).

Table 8 shows that significant effects of group were found for P1 and P2 amplitudes. Table 9 shows that significant effects of group were found for P2 and N2 latencies. These findings will be discussed in the next section.

## Discussion

The overall aim of this work was to determine the encoding of acoustic-phonetic versus phonemic representations of speech in three different language groups when the relevant cues were and were not degraded by background noise. The current findings are an extension of existing work and adds evidence to the ASP model by examining neural processing of speech in noise conditions for non-native/L2 listeners.

Interestingly, differences between groups did not interact with noise. Specifically, Hindi listeners showed larger P1 amplitudes to Hindi CV stimuli and larger P2 amplitudes for Hindi CV-V stimuli compared to the American English or Tamil listeners, regardless of noise. In addition, American English listeners showed a different N2 latency pattern for Hindi /pa/ and /pha/. Furthermore, the American English group showed the smallest P1 amplitude to the American English stimuli. In general, noise led later latency peaks for all participants and attenuation of amplitudes (except P1), but more so for the prevoiced Hindi /ba/ and the aspirated Hindi /pha/ and English /pa/.

### Morphology of the MGFP waveforms

In terms of effect of group, the morphology of the grand mean waveforms was similar across language groups (Hindi, American English, and Tamil). Even in Tamil participants, who do not have aspiration and voicing as a phonemic feature, the AEP morphology was similar to that of the other two language groups. These findings indicate that the acoustic details of voicing and aspiration were encoded at the level of AEPs. In other words, the information is encoded, even if it is not necessarily used.

In terms of the effect of noise, all of the peak responses were attenuated in noise relative to quiet, except for P1. The finding was consistent with previous literature in which smaller amplitudes or prolonged latencies were observed in noise (Martin et al., 1997, 1999; Martin and Stapells, 2005; Whiting et al., 1998; Dimitrijevic et al., 2013), except for P1 (Billings et al., 2013).

In this study, the P1 amplitudes were small in quiet and were larger in noise both for Hindi and American English stimuli. This was an interesting finding in this study because in Billings et al. (2013), the P1 amplitudes were close to the noise floor; however, in this study the P1 amplitudes were larger in noise. Although there is very little previous AEP literature to help us interpret this finding, this pattern could be due to the increased energy in the noise condition that is reflected in P1 amplitude or due to greater stimulus processing demands in noise, given that these early AEPs are obligatory in nature. This finding is in contrast to findings from Papesh et al. (2015) where reduced P1 amplitudes were noticed for 10 dB and 30 dB SNR. However, there are a couple of differences between Papesh et al.’s (2015) study and the present study: the background noise spectrum matched with the long-term spectra of speech stimuli (i.e., syllable /ba/), hence separation of signal from noise would be different and the noise was played continuously. These differences in research design would have contributed to the difference in findings across the two studies.

In contrast, P2 peak amplitudes were large in quiet and smaller in noise. This finding in the current study serves as evidence to support previous research in which P2 is an AEP component that can be closely associated with behavioral results; the reduced P2 amplitudes can be related to poorer speech perception in the presence of background noise (Billings et al., 2013).

Further, consistent with previous research (Näätänen and Picton, 1987; Hyde, 1997), N1 was found to be a better indicator for acoustic characteristics of the stimulus in this study. Additional peaks were present in the N1 region in the CV responses to Hindi /ba/ in quiet and for American English /pa/ in noise. The additional peaks in the N1 region for the CV speech sound Hindi /ba/ corresponded to prevoicing; it was also present in the subtracted waveforms, and the timing of the additional peaks corresponded to prevoicing. These findings were also consistent with the additional negative peak that was observed in some AEP research on VOTs longer than 30 ms (Sharma and Dorman, 1999). In this study, additional peaks were also observed in noise, for example, in the CV responses and the subtracted waveforms (CV - V only) to English /pa/ possibly due to the overlapping response to the vowel segment and its interaction with noise.

One interesting finding in this study that was not hypothesized was the similarity between behavioral findings and AEP results in terms of morphology of the waveforms. Patterns of perceptual assimilation related to the first language were observed in the behavioral part of the study (Antony et al., 2024): Hindi participants identified the English /pa/ as Hindi /p^h^a/ and English /ba/ as Hindi /pa/. The American English participants identified Hindi /p^h^a/ as English /pa/ and they identified both Hindi /pa/ and Hindi /ba/ as English /ba/. These patterns were predicted, given the phonetic similarity of these categories and expected assimilation patterns (Best and Tyler, 2007). Even so, accuracy was higher for the native than the non-native speech sounds. Tamil listeners identified both Hindi and English bilabial stops as one category, regardless of voicing and aspiration, as expected. Patterns of assimilation differed in quiet versus in noise.

Consistent with the behavioral results in Antony et al. (2024), the morphology in response to Hindi /p^h^a/ was similar relative to English /pa/, which is phonetically [p^h^a]. Likewise, the morphology for Hindi /pa/ was similar to English /ba/, which is phonetically [pa]. The morphology of the waveforms for Hindi /ba/ were different because of the presence of prevoicing in the stimulus. These findings add to the existing evidence that these speech sounds are primarily processed based on acoustic-phonetic representations, rather than phonemic representations.

### Peak amplitudes and latencies at FCz

An interesting finding was that effects of group were present in the central electrode site (FCz) waveforms. P1 peak amplitudes were larger in Hindi participants relative to the other language groups. Also, the P2 amplitudes were larger in native Hindi participants for Hindi stimuli relative to non-native participants (but only for the subtracted waveforms). The finding of this difference only for the subtraction, however, suggests that it is related to the consonant rather than the vowel portion. The larger P1 for Hindi participants may indicate more encoding of the acoustic-phonetic details. However, few studies have reported cross-linguistic differences in AEPs (Han et al., 2013). In terms of latency, as expected, the P2 latency was shorter in Hindi participants and N2 latency was longer in Tamil participants. Most studies to date have observed language group similarities in the P1-N1-P2 AEPs, despite phonological differences, but to date, few studies have directly examined cross-linguistic differences of AEPs (Elangovan and Stuart, 2011; Jayakumar and Narne, 2021, Sharma and Dorman, 2000). Rather, studies have focused on the MMN to non-native speech contrasts (e.g., Sharma and Dorman, 2000; Shafer, et al. 2021).

In terms of condition, AEP peak amplitudes were larger, and latencies were shorter in quiet when compared to the latencies in noise, consistent with previous literature (e.g., Dimitrijevic et al., 2013; Martin et al., 1997; Martin et al., 1999; Martin and Stapells, 2005; Whiting et al., 1998). Interactions between condition and stimuli were also present in the current study. For example, the N1, P2, and N2 peak amplitudes to English /ba/ were larger in noise, relative to the amplitudes for English /pa/. This finding illustrated that the short-lag voicing cues was less affected by noise than aspiration or prevoicing, and suggests that aspiration and prevoicing are more subtle, transient acoustic cue than (Wright, 2004).

In terms of stimulus, the Hindi /ba/ has the shortest AEP latencies, followed by Hindi /pa/ and English /ba/, with Hindi /pha/ and English /pa/ showing the longest latencies. The latencies for English /ba/ and /pa/ are consistent with other studies in which latencies were more prolonged with increases in VOT, thereby reflecting the stimulus characteristics (Elangovan and Stuart, 2011; Han et al., 2013; Sharma and Dorman, 1999; Toscano et al., 2010). However, the early latency to the Hindi prevoiced /ba/ does not match Sharma and Dorman (2000); this difference appears to be related to the method of selecting the N1 peak. Specifically, we selected the earliest negative deflection following P1. Sharma and Dorman (2000) selected a later negativity. Digeser et al. (2009) found a shorter latency N1 to a natural English /ta/ than /da/ probably because the N1 reflected the aspiration rather than the vowel onset. Other VOT studies using synthesized VOT continua did not include aspiration, resulting in a later N1. In our study, the prevoicing precedes the burst release, and thus, this stimulus shows an earlier N1 than the short-lag or long-lag aspirated stimuli because the stimuli were aligned at the burst.

Further, in this study, P2 and N2 amplitudes were relatively larger, and P1 and N1 amplitudes relatively smaller for Hindi /p^h^a/ compared to Hindi /pa/. This finding was also consistent with previous findings in which larger P2 and N2 amplitudes were more prominent to aspirated sounds when compared to unaspirated sounds. This pattern is probably related to the greater overall energy in aspirated relative to unaspirated sounds (Han et al., 2013).

Significant interactions between group and stimulus or group, stimulus and condition were also present in this study. For example, N1 morphology captured fine-grained acoustic detail, including additional peaks associated with prevoicing for Hindi /ba/. Moreover, native Hindi listeners showed evidence of more robust early encoding for aspirated stimuli (e.g., larger P1 amplitudes for /p^h^a/), consistent with greater neural synchrony for native contrasts. This finding served as additional neurophysiologic evidence to the existing literature to indicate that native listeners have better neural synchrony relative to non-native listeners, thereby supporting the ASP model (Shafer, 2024). The failure of other studies to observe this finding could possibly be related to smaller samples or differences in research design (Elangovan and Stuart, 2011; Sharma and Dorman, 2000).

While findings and interactions were often consistent with the hypotheses, there were some unexpected and interesting significant interactions as well. For example, the N2 peak latency in response to Hindi /pa/ was slightly longer relative to Hindi /p^h^a/ in American English listeners (*p* < 0.05). Also, later latency effects suggested that aspiration cues are especially vulnerable in noise for American English /pa/ Hindi /p^h^a/. N2 latencies were prolonged in noise relative to quiet, whereas some voicing-related responses showed relative stability.

Usually, one might expect longer latencies for Hindi /p^h^a/ relative to Hindi /pa/ due to its increased VOT. However, the finding in this study for the N2 from American English listeners was unexpected. The similarity in N2 latency for the Hindi /pa/ and Hindi /p^h^a/ may be related to the lower intensity of aspiration in Hindi /p^h^a/ compared to English /pa/. It is possible that without attention to the stimuli, American English participants might categorize the Hindi /p^h^a/ as English /pa/. This AEP finding was consistent with the findings from forced choice identification task in Antony et al. (2024) where the American English participants assimilated the Hindi /p^h^a/ into American English /pa/. These findings also provide the evidence to support the theoretical frameworks such as PAM-L2 (Best, 1995).

In noise, when the aspiration is masked, the group differences vanish. It is unclear whether the selected Hindi /p^h^a/ token has less aspiration than typical, although there are suggestions that Hind /p^h^a/ is characterized by breathiness rather than aspiration (Abramson and Whalen, 2017).

Another interesting finding included a significant interaction of condition and stimulus for the N2 peak latencies. The N2 latencies in response to Hindi /ba/ were similar in quiet (mean = 366 ms, s.e = 3.5) and in noise (mean = 371 ms, s.e = 3.5). However, for Hindi /pa/ and Hindi /p^h^a/, the latencies were significantly longer in noise relative to in quiet. For example, the N2 peak latency for Hindi /p^h^a/ in quiet was 414 ms (s.e = 3.524), relative to in noise 440 ms (s.e = 3.524). This finding served as a potential indicator that aspiration was difficult to process in noise, relative to prevoicing. However, given that the noise was presented each trial and not continuously makes the interpretation of the result difficult. However, this is the first ERP study to examine aspiration in quiet versus in noise; and the finding that aspiration was difficult to process relative to voicing is an important one.

### Limitations of the study and future research

Across all analyses, there was variation in the effect sizes obtained for the statistically significant findings. For example, there were several significant findings in the present study with small effect sizes (refer to the appendices for complete ANOVA tables). Only 19 findings had large effect sizes (i.e., > 0.5) and 10 findings had medium effect sizes (i.e., 0.3–0.5). Findings that had the large effect sizes were the most predictable and obvious ones, specifically, when latencies were longer for speech sounds with increased VOT (e.g., English /pa/) and in noise.

The recruitment of the Hindi and Tamil participants took place in the United States where both the Hindi and Tamil participants in this study were fluent speakers of English. Further, some of the Tamil participants had some exposure to Hindi. Analysis with and without the Tamil participants with Hindi exposure showed similar group mean values (within ±1SD), hence the data was pooled together. It is possible that some of the group differences were related to Hindi and Tamil participants being bilingual whereas many of the English participants were monolingual. Future research is required in monolingual participants. In addition, the scalp distribution from this study will be published in a separate paper.

The intermittent presentation of the noise stimuli each trial has its own limitation. Given that the noise was not played continuously throughout each run and given that while noise started at 0 ms and the burst of the five stimuli were aligned at 93 ms, a sequence of noise-onset AEP responses was likely elicited and superimposed onto the speech-evoked response on every trial. This produces morphological distortions that require further investigation. This provides the framework for our next study. In the next study, a noise-alone condition will be used to better understand if aspiration is more susceptible to noise relative to voicing and to understand how presentation of the noise (continuous versus onset at each trial) can impact these results.

Lastly, although the current findings indicate that both native and non-native listeners were able to process the speech sounds acoustic-phonetically, further research is required to examine if these findings are specific to fronto-central site or if language specific differences are reflected at temporal sites consistent with previous literature (Wagner et al., 2013). This analysis will be done in future research.

### Implications

The results of the current study have both theoretical and practical significance. This study facilitates understanding of the encoding of aspiration relative to voicing, regardless of the linguistic background. The findings from Hindi listeners served as a baseline when comparing the processing of aspiration and voicing in native Hindi listeners who have both these features with native American English listeners who have only voicing in their phonetic inventory, and native Tamil listeners who have neither.

First, the cross-linguistic similarity in AEP morphology supports a model in which cortical processing of P1-N1-P2-N2 components for stop consonant contrasts relies heavily on general auditory/acoustic-phonetic mechanisms, rather than being constrained solely by native-language phonemic categories. The presence of encoding in Tamil listeners—despite the absence of aspiration/voicing contrasts in their phonological system—suggests that cortical responses can preserve informative acoustic detail even when that detail is not consistently used for categorical decisions or in behavioral tasks (Antony et al., 2024).

Second, this work advances understanding of how noise reshapes speech-sound encoding. Peak responses were generally attenuated and/or delayed in noise relative to quiet, with the notable exception of P1, which showed larger amplitudes in noise—consistent with increased energy in noise and a reflection of the obligatory nature of P1 in encoding the increased energy in stimulus as increased P1 amplitude. In contrast, P2 amplitudes were larger in quiet and reduced in noise, supporting the interpretation that later cortical components are sensitive to the behavioral consequences of degraded speech perception. This dissociation has direct relevance for interpreting why listeners can show evidence of neural encoding even when behavioral identification declines.

Third, this study provides novel evidence regarding aspiration in adverse listening. No previous AEP study in this line of work had examined aspirated stops in background noise. Prolonged N2 latencies for American English /pa/ and Hindi /p^h^a/ in noise relative to quiet suggest that aspiration cues may be particularly susceptible to masking or cue degradation. This has implications for theories of cue weighting and robustness, as well as for understanding cross-language perceptual assimilation when cues are partially obscured.

Finally, the findings have applied implications for research and clinical contexts that involve multilingual listeners and difficult listening environments. The results indicate that neural measures can reveal preserved acoustic-phonetic encoding that may not be evident in explicit behavioral responses, supporting the use of cortical AEPs as complementary indices when studying speech processing in bilingual/multilingual populations, in individuals with limited phonological exposure, or when performance is constrained by task demands and noise.

## Conclusion

This study examined whether cortical auditory evoked potentials (AEPs) reflect primarily acoustic-phonetic encoding versus phonemic (language-specific) encoding of voicing and aspiration cues, and how this encoding changes when cues are degraded by background noise. Across Hindi, American English, and Tamil listeners, the grand-mean waveform morphology was broadly similar, including in Tamil listeners whose native phonological system does not use aspiration or voicing as contrastive features. This cross-group similarity indicates that key acoustic properties of voicing and aspiration were encoded at the cortical level even when those features were not reliably mapped to native phonemic categories.

Background noise altered cortical responses in a systematic manner. The pattern of enhanced early activity (P1) is consistent with increased energy in noise and the reduced later activity (P2) in noise is consistent with a redistribution of processing resources under degraded conditions. Together, the findings converge on the conclusion that cortical responses to voicing and aspiration are driven strongly by acoustic-phonetic representations at the midline central electrode site. However, the differences in P1 and P2 amplitude for groups are a novel finding that suggest that there may be some sharpening of encoding related to experience.

Overall, the results contribute to a better understanding of how aspiration and voicing are encoded in the cortex across listeners with different phonological inventories, and how noise modulates early stages of processing.

### Learning outcomes

1. To compare and contrast P1, N1, P2, N2
2. To identify two key findings in the study
3. To describe the acoustic-phonetic versus phonemic representation of Hindi versus English stimuli in the three language groups.

## Data Availability

The raw data supporting the conclusions of this article will be made available by the authors, without undue reservation.
